# Qishen Granules attenuate adverse ventricular remodeling in chronic heart failure by promoting Legumain/Arg1/Rac1-mediated efferocytosis of resident cardiac macrophages

**DOI:** 10.1186/s13020-026-01461-6

**Published:** 2026-07-08

**Authors:** Meijiao Tao, Shixuan Min, Jiali Liu, Huishi Xiao, Tong Xiao, Sisi Liang, Xin Liu, Jun Wang, Yulin Ouyang, Ruijia Wen, Lei Wang, Wei Wang, Yao Zhang, Chun Li

**Affiliations:** 1 Chinese Medicine Guangdong Laboratory/Hengqin Laboratory, Hengqin, 519031 Guangdong China; 2 The Second Clinical College of Guangzhou University of Chinese Medicine, Guangzhou, 510080 Guangdong China; 3 Institute of Formula and Syndrome, School of Pharmaceutical Sciences, Guangzhou University of Chinese Medicine, Guangzhou, 510006 Guangdong China; 4 State Key Laboratory of Traditional Chinese Medicine Syndrome, Guangzhou University of Chinese Medicine, Guangzhou, 510006 Guangdong China; 5 School of Basic Medical Sciences, Guangzhou University of Chinese Medicine, Guangzhou, 510006 Guangdong China; 6 School of Pharmaceutical Sciences, Guangzhou University of Chinese Medicine, Guangzhou, 510006 Guangdong China; 7 Clinical Medical College of Acupuncture Moxibustion and Rehabilitation, Guangzhou University of Chinese Medicine, Guangzhou, 510006 Guangdong China; 8 Dongguan Hospital of Guangzhou University of Chinese Medicine, Dongguan, 523000 Guangdong China; 9 Modern Research Center for Traditional Chinese Medicine, Beijing University of Chinese Medicine, Beijing, 100029 China

**Keywords:** Heart failure, Ventricular remodeling, Efferocytosis, Resident cardiac macrophages, Qishen Granules, Legumain

## Abstract

**Background:**

Ventricular remodeling is a key pathological feature of chronic heart failure (CHF). Following myocardial infarction (MI), impaired macrophage efferocytosis sustains cardiac inflammation, disrupts tissue repair, and accelerates adverse remodeling and dysfunction. Qishen Granules (QSG) is a classical traditional Chinese medicine formulation used in the clinical treatment of heart failure. However, its regulatory effects on efferocytosis in resident cardiac macrophages (RCMs) remain unclear.

**Purpose:**

This study aimed to elucidate the molecular mechanisms by which QSG regulates Legumain-mediated efferocytosis in RCMs and to identify the key bioactive compounds.

**Methods:**

A mouse model of post-MI heart failure was established via ligation of the left anterior descending coronary artery. The therapeutic effects of QSG were evaluated in isolated RCMs obtained using magnetic-activated cell sorting. Mechanistic investigations were conducted using transcriptomic analysis, network pharmacology, live-cell imaging, and siRNA-mediated gene silencing. In addition, UPLC-Q-Exactive MS, molecular docking, and molecular dynamics simulations were employed to systematically study the active compounds of QSG.

**Results:**

QSG treatment restored the population of RCMs and enhanced their efferocytic capacity in CHF, thereby reducing cardiomyocyte apoptosis, promoting inflammatory resolution, and improving ventricular structure and function. Mechanistically, QSG upregulated Legumain expression and activated the Arg1/Rac1 signaling axis to facilitate cytoskeletal remodeling and enhance phagocytic efficiency. Further analyses identified tanshinone IIA, neocryptotanshinone and calycosin as the core bioactive compounds of QSG. All three compounds upregulated Legumain expression and exhibited stable binding affinity toward Legumain.

**Conclusion:**

QSG restores efferocytic capacity in RCMs by targeting the Legumain/Arg1/Rac1 signaling axis, thereby attenuating adverse ventricular remodeling in CHF.

**Supplementary Information:**

The online version contains supplementary material available at 10.1186/s13020-026-01461-6.

## Introduction

Chronic heart failure (CHF) following myocardial infarction (MI) is a leading cause of morbidity and mortality worldwide. Currently, more than 64 million people worldwide are affected, imposing a serious threat to human health and placing a significant burden on healthcare systems [1, 2]. Ventricular remodeling after MI is a key pathological mechanism underlying the development of CHF. Although advances in interventional cardiology have markedly reduced acute mortality in patients with MI, the risk of MI-induced heart failure remains persistently high. Moreover, despite significant progress in pharmacological therapies, it remains difficult to prevent pathological ventricular remodeling entirely. Therefore, there is an urgent need to identify novel therapeutic targets and develop alternative treatment strategies to attenuate ventricular remodeling and prevent CHF.

Following MI, the rapid accumulation of necrotic and apoptotic cardiomyocytes contributes significantly to adverse ventricular remodeling and progressive cardiac dysfunction. Efferocytosis refers to the biological process by which phagocytes clear apoptotic cells (ACs). This process is essential for clearing ACs, maintaining cardiac homeostasis and attenuating inflammatory responses post-MI [3]. During MI, extensive cardiomyocyte death requires macrophages to repeatedly and rapidly engulf ACs and efficiently degrade cellular debris, thereby preventing secondary cell death and subsequent expansion of tissue necrosis. Efferocytosis involves four stages: “find-me”, “eat-me”, “engulfment”, and “post-engulfment processing”. Following internalization of ACs by macrophages, lysosomes fuse with phagosomes to form phagolysosomes. Mature phagolysosomes initiate the degradation process of ACs. Notably, arginine and ornithine derived from ACs are metabolized into putrescine by macrophage arginase 1 (Arg1) and ornithine decarboxylase [4]. Putrescine activates the actin-regulating Ras-related C3 botulinum toxin substrate 1 (Rac1) to promote cytoskeletal rearrangement and enhance phagocytosis. Therefore, in-depth investigation into alterations in cardiac macrophage efferocytosis during CHF is critical for understanding disease mechanisms and identifying new therapeutic strategies.

Macrophages are the primary professional phagocytes in the body. Cardiac macrophages exhibit high heterogeneity, and resident macrophages derived from the yolk sac possess potent phagocytic capacity. Resident cardiac macrophages (RCMs) serve as key regulators for maintaining cardiac homeostasis and promoting tissue repair after injury [5]. Under steady-state conditions, RCMs continuously clear cellular debris and damaged mitochondria from cardiomyocytes, thereby preserving mitochondrial function and metabolic balance within cardiomyocytes [6]. Emerging evidence indicates that Legumain (Lgmn), an endosomal cysteine protease highly expressed in MHC-II^low^CCR2^−^ macrophages, enhances the efferocytic capacity of RCMs. Lgmn promotes efficient engulfment and degradation of ACs, thereby facilitating cardiac repair. Clinical studies have further shown that low circulating Lgmn during the acute phase of cardiovascular disease is associated with increased mortality during follow-up [7]. Collectively, these findings underscore the critical role of RCMs-mediated efferocytosis in cardiac repair and suggest that Lgmn may serve as a therapeutic target in CHF.

Qishen Granules (QSG) is derived from the traditional Chinese medicine prescriptions “Zhen-wu” Decoction and “Si-Miao-Yong-An” Decoction. It is a well-established formula for the treatment of CHF. Our previous studies have systematically characterized its preparation process [8]. Pharmacological studies have demonstrated that QSG improves cardiac function and attenuates adverse ventricular remodeling [9, 10]. Neocryptotanshinone (NCT) and tanshinone IIA (Tan IIA), key active compounds in *Salvia miltiorrhiza*, have been shown to alleviate myocardial ischemia–reperfusion injury and enhance autophagic-lysosomal degradation [11, 12]. In addition, calycosin (CAL), a major bioactive compound of *Astragalus membranaceus*, reduces myocardial fibrosis in mice with MI by inhibiting the TGFBR1 pathway [13]. However, the role of QSG in regulating efferocytosis and modulating RCMs during ventricular remodeling remains unclear.

Based on the above, we hypothesized that QSG modulates Lgmn-mediated RCMs efferocytosis to attenuate adverse ventricular remodeling in CHF. We systematically investigated the effects of QSG on RCMs-mediated efferocytosis and its protective role in CHF using animal models, cellular experiments, transcriptomic analysis, network pharmacology, molecular docking, and genetic manipulation. We elucidated the mechanism whereby QSG inhibits ventricular remodeling and promotes cardiac repair by regulating Lgmn-mediated RCMs efferocytosis, providing new mechanistic evidence supporting the therapeutic potential of QSG in CHF.

## Materials and methods

### Drug preparation

QSG consists of *Astragalus membranaceus* (Fisch.) Bunge (Fabaceae), *Salvia miltiorrhiza* Bunge (Lamiaceae), *Aconitum carmichaelii* Debeaux (Ranunculaceae), *Scrophularia ningpoensis* Hemsl. (Scrophulariaceae), *Lonicera japonica* Thunb. (Caprifoliaceae), and *Glycyrrhiza uralensis* Fisch. ex DC. (Fabaceae). All herbal materials were purchased from Beijing Tongrentang Pharmaceutical Co., Ltd. and processed by Beijing University of Chinese Medicine. The batch of QSG used in this experiment is identical to that used in the related published study [14]. Fosinopril sodium tablets were used as a positive control (H19980197, Bristol-Myers Squibb Pharmaceutical Co., Ltd., China).

### QSG component analysis based on UPLC-MS/MS

QSG (80 mg) was thawed at 4 °C and extracted with 1 mL water-acetonitrile-isopropanol (1:1:1, v/v/v) by homogenization and low-temperature ultrasonication, followed by centrifugation. The supernatant was then subjected to protein-precipitated, re-centrifuged, vacuum-dried, and reconstituted in 200 μL 30% acetonitrile, followed by a final centrifugation prior to analysis. UPLC-MS/MS analysis was performed as previously described [15].

### Animals

Twenty 10-week-old male Sprague–Dawley (SD) rats and 186 8-week-old male ICR mice were purchased from Guangdong Zhiyuan Biomedical Technology Co., Ltd. (Guangdong, China). The animal production license number is SCXK (Yue) 2021–0057. They were housed in the specific pathogen-free system of the Laboratory Animal Center, License No.: SYXK (Yue) 2024–0202. All mice were included in subsequent experiments, covering pharmacodynamic evaluation, TTC staining, molecular biological assays of primary RCMs, flow cytometry, temporal profiling post-MI, transcriptomic sequencing, and in vitro functional experiments of RCMs. Given distinct experimental endpoints and sample collection requirements, separate animal cohorts were used for each assay. Experimental animals were euthanized by carbon dioxide inhalation. The sample size of each group in all experiments has been clearly specified in the corresponding figure legends.

### Surgical procedures to induce HF

A heart failure model was established by ligation of the left anterior descending (LAD) coronary artery, as previously described [16]. In the sham group, the same surgical procedure was performed without ligation. Surviving mice were randomly divided into five groups: sham group, model group, low-dose QSG (QSG-L, 1.42 g/kg/d) group, high-dose (QSG-H, 5.66 g/kg/d) group, and fosinopril group. The QSG dosage was determined based on the clinically equivalent dose. The positive control group received fosinopril at 15 mg/kg/d. The sham and model groups received saline. All treatments were dissolved in saline and administered by oral gavage from days 7 to 14 after successful model establishment.

### Echocardiographic assessment

Echocardiographic measurements were performed under light anesthesia using a high-resolution ultrasound imaging system (Vevo 2100, FUJIFILM VisualSonics, Canada). Two-dimensional M-mode images at the level of the papillary muscles were used to assess left ventricular structure and function. The measured parameters included left ventricular (LV) ejection fraction (LVEF), fractional shortening (LVFS), anterior wall diastolic thickness (LVAW;d), anterior wall systolic thickness (LVAW;s), posterior wall diastolic thickness (LVPW;d), posterior wall systolic thickness (LVPW;s), end-diastolic internal dimension (LVID;d), and end-systolic internal dimension (LVID;s).

### Histopathological examination

Mouse hearts were rinsed with cold PBS, frozen at −80 °C, and uniformly cross-sectioned into 5–7 slices at the ligation level. TTC staining was performed according to the manufacturer’s instructions (T8877, Sigma-Aldrich, USA). Heart tissue was fixed in paraformaldehyde and sectioned, stained with HE (G1120, Servicebio, Wuhan, China) and Masson’s trichrome (G1340, Servicebio, Wuhan, China). Pathological sections were examined under a whole-slide scanning system (VS200, OLYMPUS, Japan). Histopathological analyses were analyzed using ImageJ (Fiji, NIH, USA).

### Serum marker detection

Blood samples were collected from the retro-orbital plexus and allowed to clot at room temperature for 1 h. Samples were then centrifuged to obtain serum. LDH (A020-2, Jiancheng, Nanjing, China) levels were measured using a microplate-based assay. BNP (BY-EM220519, Byabscience, Nanjing, China) and cTnI (BY-EM220716, Byabscience, Nanjing, China) levels were detected via ELISA according to the manufacturers' instructions. Absorbance values were measured using a Hybrid Multi-Mode Microplate Reader (Synergy H1, BioTek, USA).

### RNA isolation and quantitative real-time PCR (RT-qPCR)

Total RNA was extracted from tissues and cells, and reverse-transcribed into cDNA according to the manufacturer’s instructions (EZB-RN001-plus, A0010CGQ, A0012-R2, EZBioscience, China). Quantitative real-time PCR was then performed. The relative expression of genes was calculated using the 2^−△△CT^ method with GAPDH as the internal reference gene, and the sequences of primers were shown in Table 1.

**Table 1:** List of primers of the genes

Gene	Sense	Antisense
*Bax*	CGTGGTTGCCCTCTTCTACTTTG	TCCAGTGTCCAGCCCATGATG
*Bcl2*	CCCCTGGCATCTTCTCCTTCC	ATGGACCACAGGTGGCACAG
*Tim4*	CATTGCCTGCTGTGTGGGATTTG	TGTCATTGAGGACGCTGTCACTATC
*Cx3cr1*	CGGTCTGGTGGGAAATCTGTTG	AGGTTCAGGAGGTAGATGTCAGTG
*S1p*	CTGACCTTCCGCAAGAACATCTCC	CCCAGCAGGCAATGAAGACACTC
*Arg1*	CAGAAGAATGGAAGAGTCAGTGTGG	GGAGTGTTGATGTCAGTGTGAGC
*Rac1*	CCGCAGACAGTTGGAGACACATG	ATGCAGGACTCACAAGGGAAAAGC
*Mfge8*	AGGAGCAAGGAAGCAGCAAGG	TGATGCGGTTATGCCAGGACAC
*Legumain*	GCCTGCTACTACGACGAGGAGAG	CTGGTGTTGGTGTGGGACTTGAC
*Gapdh*	GGTTGTCTCCTGCGACTTCA	TGGTCCAGGGTTTCTTACTCC

### Western blot

Magnetically sorted cells and tissues were lysed in RIPA (P0013B, Beyotime, China). Western blotting was performed as described previously [7]. The following primary antibodies were used: Caspase-3 Antibody (#9622, 1:1000, Cell Signaling Technology, USA), Cleaved caspase-3 Antibody (#9661, 1:1000, Cell Signaling Technology, USA), GAPDH (AF7021, 1:5000, Affinity, China), Legumain (CY8236, 1:1000, Abways, China), Arg1 (ET1605-8, 1:1000, HUABIO, China), and Rac1 (#21201, 1:500, Signalway Antibody, USA). Protein bands were visualized using an enhanced chemiluminescence reagent (SQ201, Epizyme, China).

### Immunofluorescence staining and terminal deoxynucleotidyl transferase-mediated dUTP nick-end labeling (TUNEL) assay

Immunofluorescence staining was performed as previously described [17]. Samples were incubated with the following primary antibodies: CD68 Ab (AB3506, 1:200, Abways, China), Legumain (LGMN) Rabbit mAb (A23776, 1:200, ABclonal, China), Liver Arginase Recombinant Rabbit Monoclonal Antibody [SY09-06] (ET1605-8, 1:500, HUABIO, China), cTnI antibody (ab47003, 1:200, Abcam, USA), and PE Rat Anti-Mouse F4/80 (565410, 1:200, BD Biosciences, USA). Subsequently, the corresponding secondary antibodies: Donkey Anti-Mouse IgG H&L (Alexa Fluor 488) (ab150105, 1:200, Abcam, USA), Donkey anti-Rabbit IgG (H + L) Highly Cross-Adsorbed Secondary Antibody, Alexa Fluor 555 (A-31572, 1:200, Thermo Fisher Scientific, USA) and Donkey Anti-Rabbit IgG H&L (Alexa Fluor 647) (ab150075, 1:500, Abcam, USA) were applied and incubated at room temperature for 2 h. Nuclei were counterstained with DAPI-containing antifade mounting medium (MA0222, Meilunbio, Dalian, China). TUNEL staining was performed with an In Situ Apoptosis Detection kit (E-CK-A321, Elabscience, China). Fluorescence images were acquired using a confocal microscope (Olympus IXplore SpinSR, Japan).

### Preparation of drug-containing serum

Ten Sprague–Dawley (SD) rats were administered QSG at a dose of 3.92 g/kg/day, while ten rats in the control group were given an equal volume of sterile water. The preparation of QSG drug-containing serum was performed as previously described [18].

### Isolation and culture of primary cardiomyocytes and RCMs

Primary cardiomyocytes were isolated from neonatal mice. The left ventricles were excised and minced in PBS, then digested with 0.1% collagenase II (17101015, Gibco, USA) at 37 °C with gentle agitation for 30 min. The cell suspension was filtered through a 70 μm strainer. Subsequently, cells were seeded at a density of 2 × 10^6^ cells per dish and cultured in cardiomyocyte culture medium supplemented with 10% FBS (10270106, Gibco, USA) and 0.1 mM 5-bromo-2’-deoxyuridine (B9285, Sigma-Aldrich, USA) for 3–5 days.

For isolation of RCMs, hearts were minced and enzymatically digested with collagenase II and DNase I (60 U/L, R017414, Rhawn, China) at 37 °C for 30 min. The single-cell suspension was incubated with F4/80 MicroBeads (130-110-443, Miltenyi Biotec, Germany). Subsequently, magnetic separation was performed, and F4/80-positive cells within the separation column were collected.

### Induction of apoptosis in cardiomyocytes

Cardiomyocyte apoptosis was induced by ultraviolet (UV) irradiation (254 nm) for 7 min, followed by incubation at 37 °C for 3 h.

### Cell counting kit-8 (CCK-8) assay

RCMs were seeded into 96-well plates at an appropriate density and incubated for 3–5 days to facilitate cell adhesion. Cell viability was assessed using the CCK-8 (CA1210, Solarbio, China) according to the manufacturer’s instructions.

### Flow cytometry analysis

Cardiac single-cell suspensions were first incubated with anti-CD16/32 antibody (FMU16/32-02-100, 4A BIOTECH, China) to block Fc receptors. The following antibodies were used for flow cytometry: CD45-APC-Cy7 (557659, BD Biosciences, USA); F4/80-PE (565410, BD Biosciences, USA); CD11b-PerCP-Cy5.5 (550993, BD Biosciences, USA); CD192 -Brilliant Violet 510 (150617, BioLegend, USA); I-A/I-E -Alexa Fluor 647 (562367, BD Biosciences, USA); Legumain-Alexa Fluor 594 (sc-433234, Abcam, USA); cTnI antibody (ab47003, Abcam, USA); Donkey Anti-Rabbit IgG H&L (Alexa Fluor 647) (ab150075, Abcam, USA); and Fixable Viability Stain 700 (564997, BD Biosciences, USA). Flow cytometry was performed using a BD LSRFortessa flow cytometer (BD Biosciences, USA), and data were analyzed with FlowJo 10.8.1 software.

### Live cell imaging

RCMs were seeded in confocal dishes at a density of 1 × 10^4^ cells per dish. After adhesion, RCMs were stained using the Cell Tracking Dye Kit-Green (ab138891, Abcam, USA) at 37 °C for 15 min. Primary cardiomyocytes were seeded at a density of 5 × 10^4^ cells per 10 cm dish. Following apoptosis induction, the cardiomyocytes were stained with the Cell Tracking Dye Kit-Deep Red (ab138894, Abcam, USA) at 37 °C for 15 min. ACs were then co-cultured with RCMs at a ratio of 5:1 (ACs: RCMs). Live-cell imaging was performed using a spinning-disk confocal microscope. Images were acquired every 30 s for a total duration of 2 h.

### Small interfering RNA (siRNA) transfection

Three siRNA sequences targeting murine *Lgmn* were synthesized by Hanheng Biotechnology (Shanghai, China). Among them, sequence 3 showed the highest knockdown efficiency and was selected for subsequent experiments. The sequences were as follows: GACUAUUCGCUACAUGUAUGA (sense) and AUACAUGUAGCGAAUAGUCUU (antisense). The sequences of negative control siRNA (nc-siRNA) were UUCUCCGAACGUGUCACGU (sense) and ACGUGACACGUUCGGAGAATT (antisense). RCMs were seeded in 12-well plates and cultured for 3–5 days. Cells were then transfected with 50 nM Lgmn siRNA or 50 nM nc-siRNA using 6.25 μL of RNA Fit transfection reagent (Hanheng Biotechnology, Shanghai, China). RT-qPCR was performed 24 h post-transfection to evaluate knockdown efficiency. Subsequent experiments were conducted at 24 h after transfection.

### Network pharmacology analysis

The six herbal components of QSG were individually queried in the Traditional Chinese Medicine Systems Pharmacology Database and Analysis Platform (TCMSP). Active compounds were screened based on oral bioavailability ≥ 30% and drug-likeness ≥ 0.18. CHF targets were collected from the GeneCards and OMIM databases, and 382 efferocytosis-related genes were retrieved from the GeneCards, Gene Ontology (GO), and Kyoto Encyclopedia of Genes and Genomes (KEGG) databases. Common targets were identified using Venn diagram analysis and subsequently used to construct a protein–protein interaction (PPI) network. The network was visualized and analyzed using Cytoscape v3.10.2. Enrichment results with *P* < 0.05 were considered statistically significant and visualized.

### Transcriptome sequencing

Total RNA was extracted from mouse RCMs and RNA quality was assessed using an Agilent 2100 Bioanalyzer (Agilent Technologies, CA, USA). Sequencing libraries were constructed and amplified by PCR, followed by sequencing on an Illumina platform. Differentially expressed genes (DEGs) were identified using DESeq2 with the following criteria: *P* < 0.01 and |log_2_FC|≥ 0.4. Subsequently, enrichment analyses were performed.

### Molecular docking and molecular dynamics simulations (MDS)

The three-dimensional structure of LGMN was obtained from the RCSB Protein Data Bank (PDB), and ligand structures were retrieved from the ChemSpider database. Protein structure was prepared and visualized using PyMOL (version 2.5). Molecular docking was performed using AutoDock 4, using ADT for grid parameter definition and LGA for conformational search. The docked conformations were visualized and analyzed using PyMOL.

Molecular dynamics simulations were performed using the docked small molecule-protein complexes as the initial configurations, employing AMBER 24 software [19]. The binding free energies between proteins and ligands were calculated using the MM/GBSA method [20].

### Statistical analysis

Statistical analyses were performed using GraphPad Prism software. All data are presented as mean ± standard error of the mean (SEM). An unpaired two-tailed t-test was used for comparison between groups. For comparisons among three or more groups, one-way ANOVA was used (followed by LSD or Dunnett’s multiple comparison test) when data met the assumptions of normality and homogeneity of variance; otherwise, non-parametric tests were applied. Post hoc power analysis was performed in selected experiments using G*Power software, based on the observed effect sizes and a significance level of α = 0.05, to calculate the statistical power for each comparison, thereby assessing the adequacy of the current sample size. A *P* value < 0.05 was considered statistically significant.

## Results

### Quality control of QSG

Our group previously performed quantitative analyses of the absorbed components of QSG in rat plasma [21, 22]. In the present study, the components of QSG were further characterized using UPLC-MS/MS. A total of 11 compounds were identified, including calycosin, isoliquiritigenin, ononin, glycyrrhizic acid ammonium salt, tanshinone IIA, salvianolic acid B, glycyrrhizic acid, isochlorogenic acid C, neochlorogenic acid, sweroside, and harpagide (Fig. S1).

### QSG improves cardiac function and attenuates adverse ventricular remodeling in mice with MI-induced CHF

**Fig. 1:**
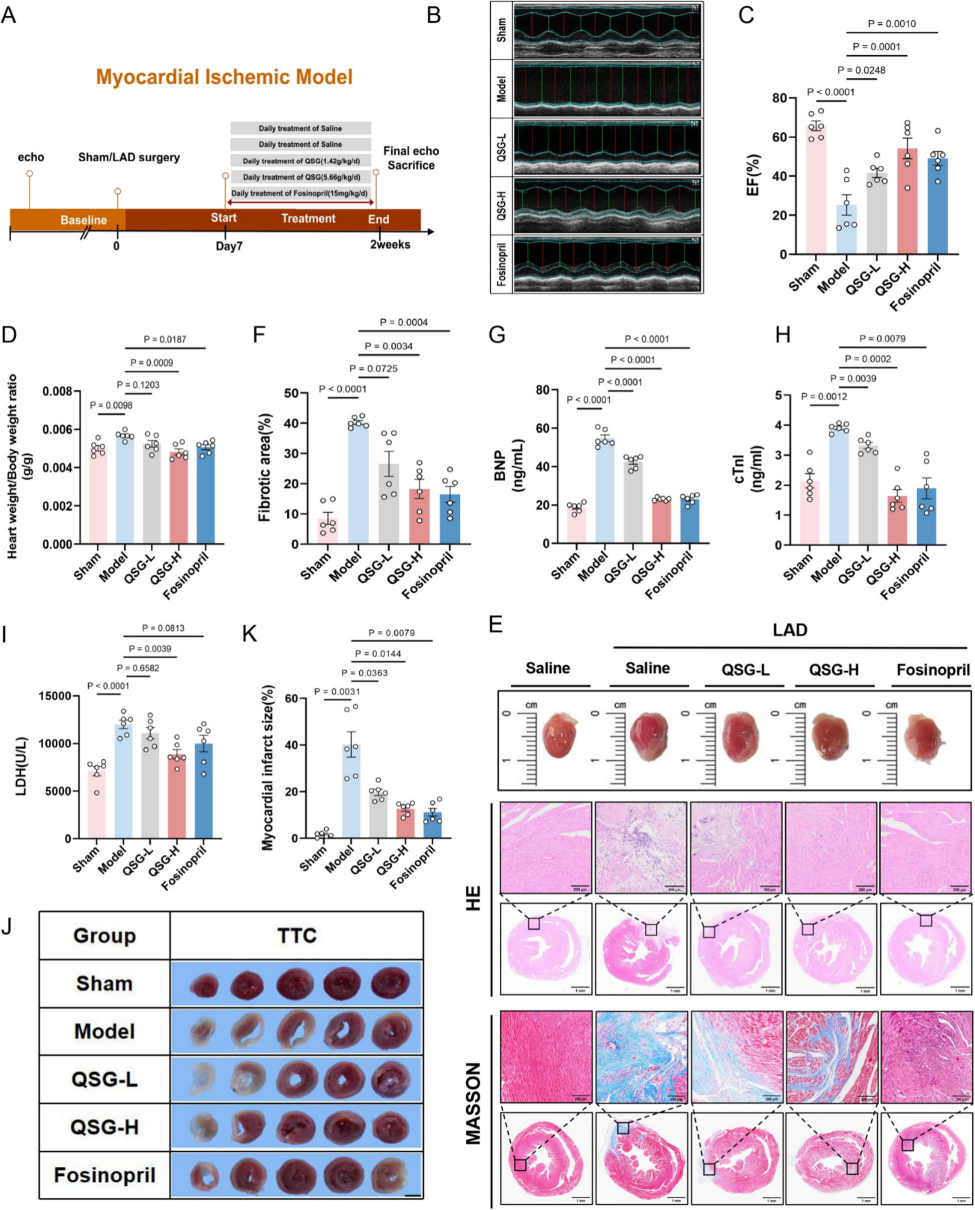
The therapeutic effects of QSG in mice with post-MI heart failure. **A** Experimental design of the animal study. **B** Representative M-mode echocardiographic images from each group. **C** Quantitative analysis of EF measured by echocardiography (n = 6). **D** HW/BW ratio in each group (n = 6). **E** Representative macroscopic heart images, HE staining, and Masson’s trichrome staining in each group. **F** Quantitative analysis of myocardial fibrosis based on Masson's trichrome staining (n = 6). **G** Serum BNP levels in each group (n = 6). **H** Serum cTnI levels in each group (n = 6). **I** Serum LDH levels in each group (n = 6). **J** Representative TTC-stained images of heart from each group. **K** Quantitative analysis of myocardial infarction area in each group (n = 6). n indicates the number of experimental animals per group. All data are presented as mean ± SEM

The progression of post-MI heart failure exhibits distinct temporal characteristics. Previous studies have indicated that days 7–14 after MI represent a critical window for cardiac repair, during which the inflammatory storm gradually subsides but substantial clearance of apoptotic and necrotic cells remains necessary [23]. This stage also corresponds to the typical intervention period for traditional Chinese medicine in clinical practice. To evaluate the therapeutic effect of QSG on CHF, a mouse model of ischemic heart failure was established by ligation of the LAD coronary artery. From day 7 to day 14 after MI induction, mice were administered different doses of QSG or fosinopril daily by gavage (Fig. 1A). Echocardiographic analysis revealed that the model group developed significant cardiac dysfunction compared with the sham group, as evidenced by reduced EF and FS. Additional cardiac function parameters are presented in Fig. S2. Compared with the model group, QSG significantly improved EF in a dose-dependent manner, with effects comparable to the positive control fosinopril (Fig. 1B, C). In addition, both QSG and fosinopril significantly reduced the elevated heart weight-to-body weight (HW/BW) ratio (Fig. 1D). HE and Masson staining demonstrated that QSG ameliorated myocardial structural disruption and reduced cardiac fibrosis (Fig. 1E, F). Serological analyses further showed that both QSG and fosinopril decreased the levels of BNP, as well as the myocardial injury markers cTnI and LDH (Fig. 1G–I). Furthermore, TTC staining revealed that both QSG and fosinopril effectively reduced myocardial infarct size after MI (Fig. 1J, K). Taken together, these findings demonstrate that QSG effectively improves cardiac function and attenuates adverse ventricular remodeling in mice with MI-induced heart failure.

### QSG reduces cardiomyocyte apoptosis in CHF mice

**Fig. 2:**
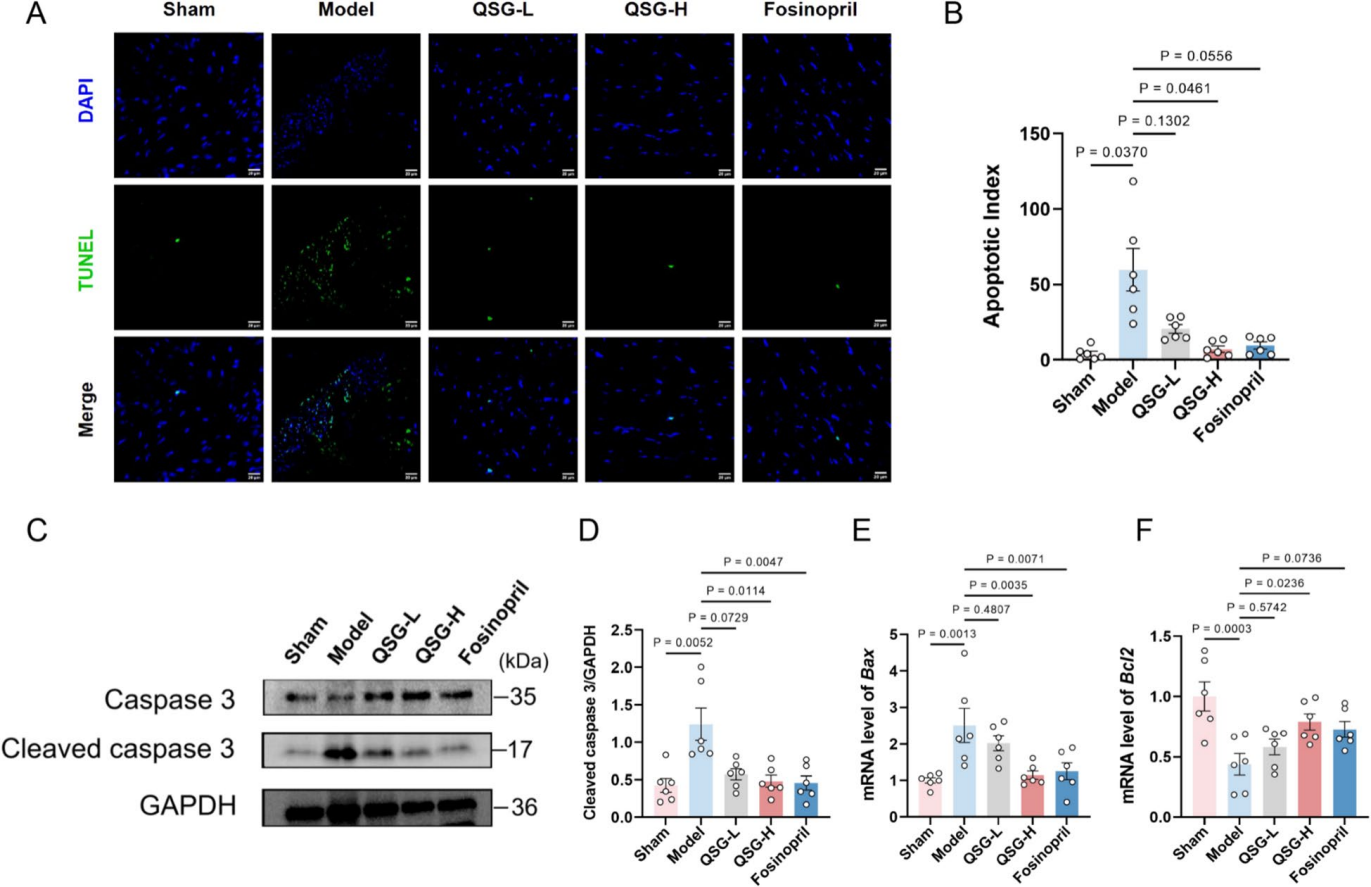
Protective effect of QSG on cardiomyocytes apoptosis in mice with heart failure. **A** TUNEL staining of heart from each group, apoptotic nuclei (green), cardiomyocyte nuclei (blue), scale bar: 20 μm. **B** Quantitative analysis of TUNEL-positive cells in each group (n = 6). **C** Representative Western blot images of Cleaved Caspase-3 in the cardiac infarct area of mice from each group. **D** Quantitative analysis of Cleaved caspase-3 protein expression (n = 6). **E**, **F** mRNA expression levels of *Bax* and *Bcl-2* in the cardiac infarct area of mice from each group (n = 6). n indicates the number of experimental animals per group. All data are presented as mean ± SEM

To explore the potential mechanism underlying the therapeutic effects of QSG in CHF, we first performed a network pharmacology analysis. A total of 1,247 QSG-related targets were identified from the TCMSP database, while 1,353 heart failure-associated targets were retrieved from the GeneCards and OMIM databases. By intersecting these datasets, 163 common targets were identified as candidate core targets (Fig. S3A). Subsequently, KEGG enrichment analysis was performed to predict the potential mechanisms underlying the effects of QSG in CHF. The results showed that the key signaling pathways involved pathways related to apoptosis, autophagy, efferocytosis and lysosomal-related pathways (Fig. S3B). To validate these predictions, we performed a TUNEL assay. The results demonstrated that the number of ACs in the infarcted myocardium was significantly increased in CHF mice compared with the sham group. Treatment with QSG or fosinopril markedly reduced the number of ACs (Fig. 2A, B). In addition, QSG significantly decreased the protein level of cleaved caspase-3 and the mRNA levels of *Bax*, while upregulating *Bcl-2* mRNA level (Fig. 2C–F). These findings indicate that QSG effectively attenuates cardiomyocyte apoptosis in CHF.

### Integrated transcriptomic and network pharmacology analyses of RCMs reveal that QSG regulates Lgmn/Arg1/Rac1-mediated efferocytosis in CHF

**Fig. 3:**
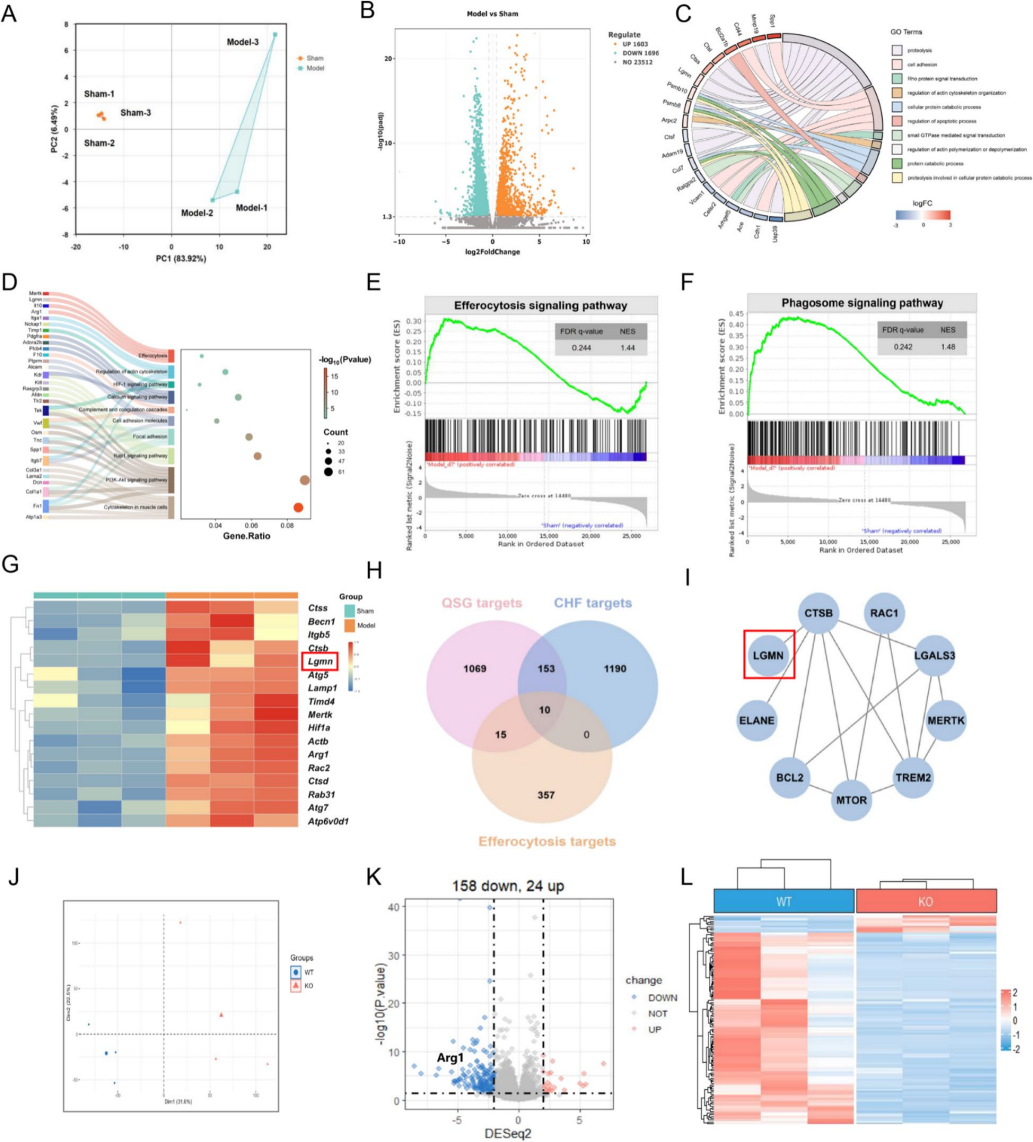
Integrated transcriptomics and network pharmacology analysis of RCMs in CHF mice treated with QSG. **A** Principal component analysis (PCA) of the Sham and Model groups (n = 3). **B** Volcano plot of differential genes between Sham and Model groups. **C** GO enrichment analysis of DEGs in Sham and Model groups. **D** KEGG enrichment analysis of DEGs in Sham and Model groups. **E**, **F** GSEA of efferocytosis and phagosome signaling pathways in Sham and Model groups. **G** Heatmap of efferocytosis-related DEGs between Sham and Model groups. **H** QSG, CHF, and efferocytosis targets cross analysis. **I** PPI network based on intersecting targets shown in (H). **J** PCA of cardiac macrophages from WT and Lgmn^⁻/⁻^ mice after MI. **K** Volcano plot of DEGs in cardiac macrophages from WT and Lgmn^⁻/⁻^ mice post-MI (FDR ≤ 0.05, fold change ≥ 2). **L** Heatmap of DEGs in cardiac macrophages from WT and Lgmn^⁻/⁻^ mice after MI. n indicates the number of experimental animals per group

To further investigate the molecular mechanisms underlying CHF progression, transcriptome sequencing was performed on RCMs isolated from mice at 7 days post-MI (Fig. 3A, B). GO analysis revealed significant enrichment in biological processes related to efferocytosis, including regulation of actin cytoskeleton organization, regulation of apoptotic process, and cell adhesion (Fig. 3C). KEGG analysis further revealed significant enrichment of the efferocytosis pathway, cytoskeleton in muscle cells, and Rap1 signaling pathway (Fig. 3D). Gene set enrichment analysis (GSEA) also showed significant enrichment in efferocytosis and phagocytic signaling pathways (Fig. 3E, F). These findings suggest that efferocytosis plays a critical role in the progression of CHF. Among the DEGs, Lgmn was significantly altered and occupied a central position within the efferocytosis, indicating its potential importance in regulating efferocytosis during CHF (Fig. 3G). Notably, RT-qPCR validation confirmed the transcriptomic findings, demonstrating that Lgmn expression was significantly increased at 7 days post-MI (Fig. S4A).

To further identify key therapeutic targets of QSG, we performed an integrated analysis of QSG targets, heart failure targets, and efferocytosis-related targets. Ten overlapping targets were identified, including Lgmn (Fig. 3H, I). Topological analysis of the PPI network showed that Lgmn has a degree value of 1, a betweenness centrality of 0, and a closeness centrality of 0.47. These results indicate that QSG may exert its cardioprotective effects by targeting Lgmn to regulate the efferocytosis signaling pathways. To further clarify the downstream regulatory mechanism of Lgmn, we analyzed transcriptomic data from cardiac macrophages derived from WT and Lgmn^⁻/⁻^ mice after MI in the GEO database (GSE198735). A total of 182 differentially expressed genes were identified, including 158 downregulated genes and 24 upregulated genes in Lgmn^−/−^ mice compared with WT. Notably, Arg1 expression was significantly reduced in Lgmn^−/−^ mice (Fig. 3J–L). GO analysis indicated that Lgmn is associated with various biological processes including leukocyte adhesion and cell chemotaxis (Fig. S4B), while KEGG enrichment analysis revealed significant enrichment in the cytokine-cytokine receptor interaction pathway and chemokine signaling pathway (Fig. S4C). Given that the Arg1/Rac1 axis constitutes a well-established regulatory pathway involved in efferocytosis, these findings suggest that Lgmn may mediate macrophage efferocytosis by modulating Arg1/Rac1 signaling, thereby facilitating the clearance of ACs and contributing to cardiac repair after MI. Moreover, these results provide mechanistic evidence that QSG may alleviate ventricular remodeling by targeting Lgmn to regulate the Arg1/Rac1 axis and enhance efferocytosis.

### QSG enhances efferocytosis in RCMs in mice with heart failure

**Fig. 4:**
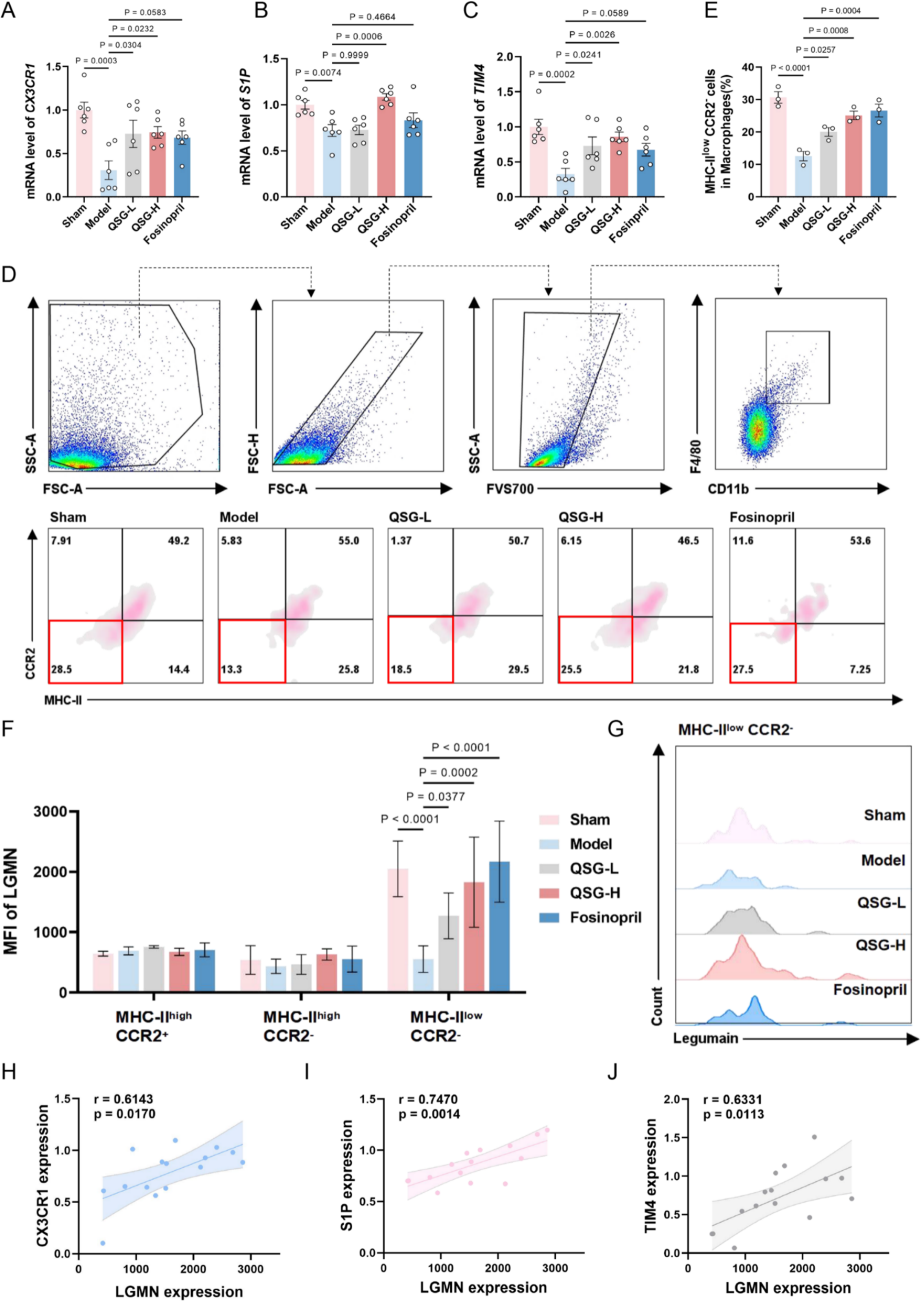
Protective effect of QSG on RCMs efferocytosis in mice with heart failure. **A**–**C** mRNA expression levels of *S1p*, *Tim4*, and *Cx3cr1* in the cardiac infarct area of mice from each group (n = 6). **D** Gating strategy for flow cytometric analysis of macrophage subsets in cardiac tissues from each group. **E** Quantification of MHC-II^low^CCR2^−^ macrophages in mouse hearts from each group (n = 3). **F** Quantitative analysis of LGMN mean fluorescence intensity (MFI) in different macrophage subsets from each group (n = 3). **G** Flow cytometric histograms showing LGMN expression in MHC-II^low^CCR2^−^ macrophage subsets from each group. **H**–**J** Correlation analysis between LGMN and *Cx3cr1*, *S1p*, and *Tim4* expression. n indicates the number of experimental animals per group. All data are presented as mean ± SEM

We first evaluated the mRNA expression of key efferocytosis-related genes, including *S1P*, *Tim4*, and *Cx3cr1*, in cardiac tissues. Compared with the sham group, the expression levels of these genes were significantly reduced in CHF mice (Fig. 4A–C). Notably, QSG treatment markedly reversed these reductions, suggesting that QSG may improve efferocytosis by enhancing ACs recognition and macrophage recruitment, thereby alleviating adverse ventricular remodeling. Flow cytometry analysis was subsequently performed to assess cardiac macrophage populations. The proportion of MHC-II^low^CCR^−^ resident macrophages with strong phagocytic ability was significantly reduced in CHF mice. QSG treatment significantly restored the proportion of this macrophage subset (Fig. 4D, E). Furthermore, we examined Lgmn expression in different cardiac macrophage subsets. Lgmn was predominantly expressed in MHC-II^low^CCR2^−^ macrophages. Its expression was markedly reduced in CHF mice but was significantly upregulated by QSG treatment (Fig. 4F, G). To further explore the underlying mechanistic association, we analyzed the correlations between Lgmn MFI in RCMs and the mRNA expression of core efferocytosis-related genes including *CX3CR1*, *S1P* and *TIM4* using matched cardiac tissue samples. The results demonstrated that Lgmn expression was significantly positively correlated with these key efferocytosis-associated markers, suggesting that increased Lgmn expression is closely associated with enhanced efferocytosis-related functional activity of RCMs (Fig. 4H–J). In conclusion, these findings indicate that QSG enhances efferocytosis in RCMs, potentially through the upregulation of Lgmn expression.

### QSG ameliorates heart failure by modulating the Lgmn/Arg1/Rac1 signaling pathway

**Fig. 5:**
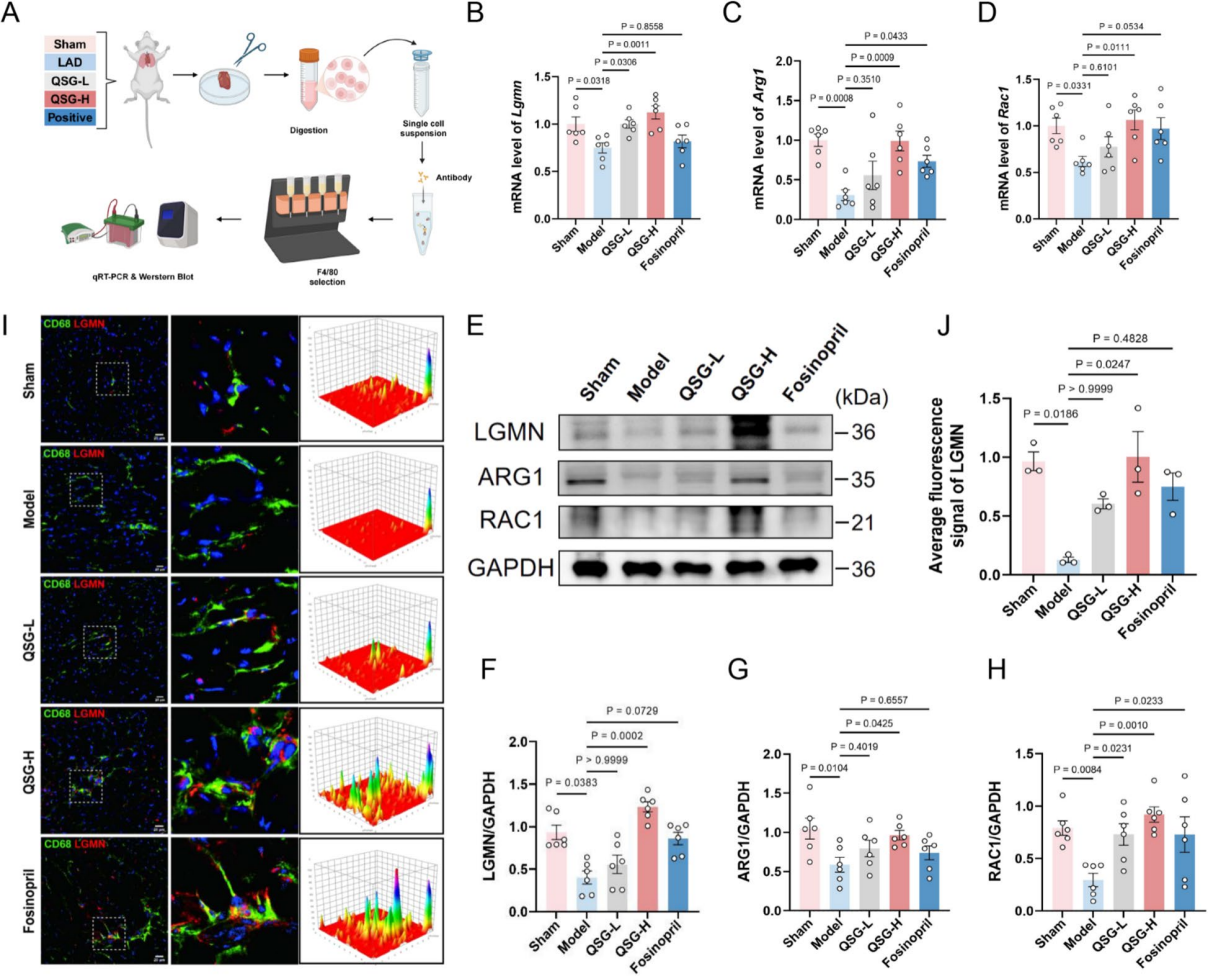
QSG ameliorates heart failure by modulating the Lgmn/Arg1/Rac1 signaling pathway. **A** Schematic diagram of the experimental protocol. **B**–**D** mRNA expression levels of *Lgmn*, *Arg1*, and *Rac1* in RCMs from each group (n = 6). **E** Representative Western blot images of LGMN, ARG1 and RAC1 protein expression. **F**–**H** Quantitative analysis of LGMN, ARG1 and RAC1 protein expression in RCMs in each group (n = 6). **I** Representative immunofluorescence images in cardiac infarct area of mice from each group, CD68 (green), LGMN (red), scale bar: 20 μm. **J** Quantitative analysis of LGMN fluorescence intensity in cardiac tissues from each group (n = 3). n indicates the number of experimental animals per group. All data are presented as mean ± SEM

Cardiac macrophages were isolated from mice following 7 days of treatment using magnetic bead sorting. The mRNA and protein expression levels of Lgmn, Arg1, and Rac1 were subsequently analyzed by RT-qPCR and Western blot, respectively (Fig. 5A). Treatment with both QSG and fosinopril significantly reversed the downregulation of Lgmn, Arg1, and Rac1 expression in cardiac macrophages (Fig. 5B–H). In addition, immunofluorescence analysis of CD68 and Lgmn showed that both QSG and fosinopril significantly increased Lgmn expression in cardiac macrophages from CHF mice (Fig. 5I, J). Taken together, these findings suggest that QSG enhances the efferocytic capacity of RCMs by modulating the Lgmn/Arg1/Rac1 signaling pathway, thereby reducing ACs accumulation and attenuating adverse ventricular remodeling in heart failure.

### QSG enhances efferocytosis of RCMs by targeting the Lgmn/Arg1/Rac1 signaling pathway

**Fig. 6:**
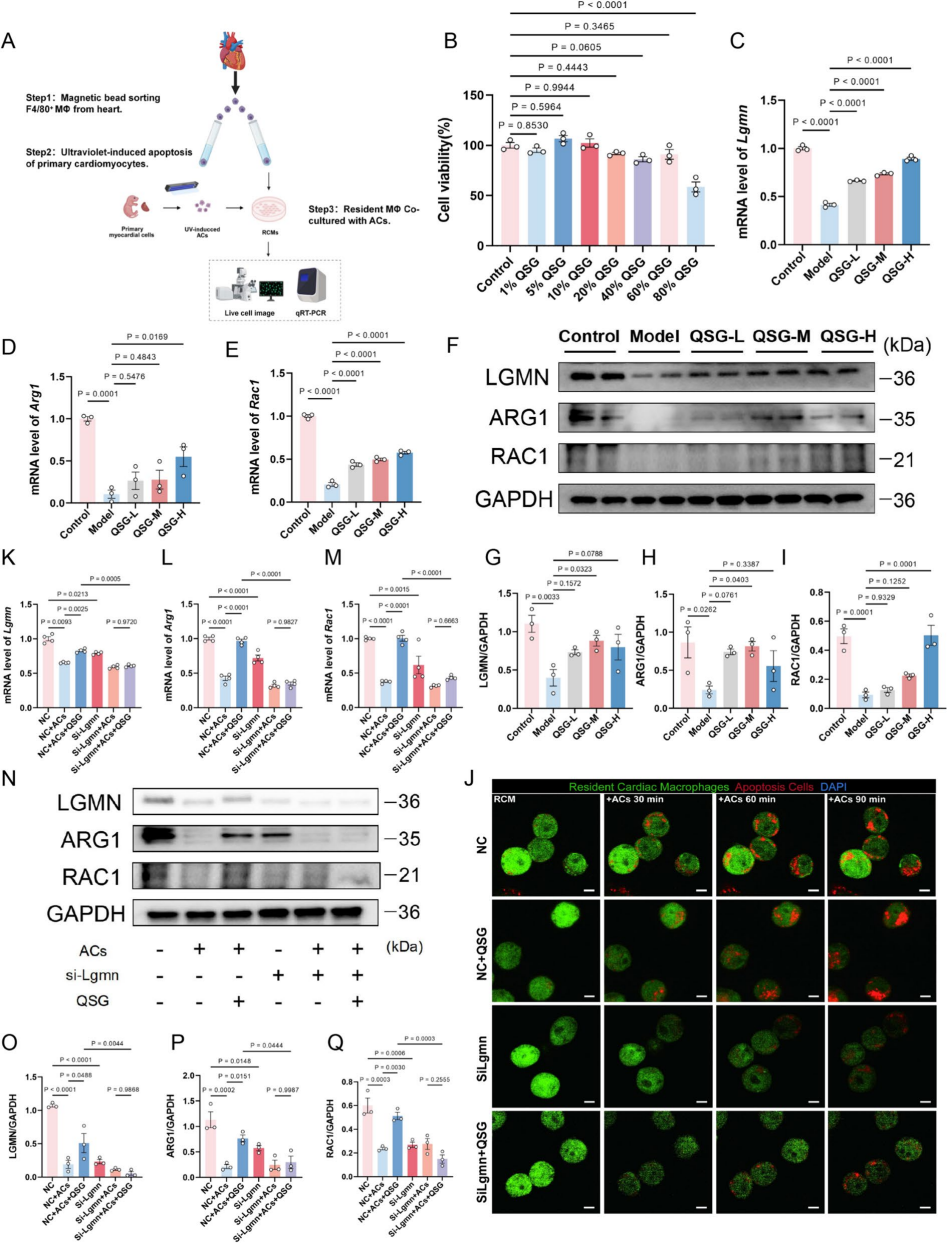
QSG enhances efferocytosis of RCMs by targeting the Lgmn/Arg1/Rac1 signaling pathway. **A** Schematic diagram of the experimental procedure. **B** Cell viability of RCMs following QSG treatment (n = 3). **C**–**E** mRNA expression levels of *Lgmn*, *Arg1*, and *Rac1* in RCMs-ACs co-culture system following QSG treatment (n = 3). **F** Representative Western blot images of LGMN, ARG1 and RAC1 protein expression. **G**–**I** Quantitative analysis of LGMN, ARG1 and RAC1 protein expression in RCMs from each group (n = 3). **J** Live-cell imaging of RCMs-ACs co-culture system under different treatments, RCMs (green), ACs (red), scale bar: 5 μm. **K**–**M** mRNA expression levels of *Lgmn*, *Arg1*, and *Rac1* in Lgmn-knockdown RCMs-ACs co-culture system following QSG treatment (n = 4). **N** Representative Western blot images of LGMN, ARG1 and RAC1 protein expression in Lgmn-knockdown RCMs-ACs co-culture system following QSG treatment. **O**–**Q** Quantitative analysis of LGMN, ARG1 and RAC1 protein expression in Lgmn-knockdown RCMs-ACs co-culture system following QSG treatment (n = 3). n represents the number of independent experiments. All data are presented as mean ± SEM

RCMs were isolated from normal mouse hearts via magnetic bead sorting. while Primary cardiomyocytes were isolated from neonatal mouse hearts and treated with ultraviolet irradiation to induce apoptosis (Fig. S5A-D). The CCK-8 assay confirmed that the QSG-containing serum at concentrations below 60% exhibited no cytotoxic effects on RCMs (Fig. 6B). Subsequently, RCMs were co-cultured with ACs. Time-course analysis revealed that *Lgmn* mRNA expression was significantly reduced after 24 h of co-culture with ACs (Fig. S6A). To evaluate the effect of QSG on RCMs efferocytosis, the RCMs-ACs co-culture system was treated with 5%, 10%, or 20% QSG-containing serum. QSG dose-dependently promoted the mRNA and protein expression levels of Lgmn, Arg1, and Rac1, suggesting that QSG may enhance the efferocytic capacity of RCMs through activation of the Lgmn/Arg1/Rac1 signaling pathway (Fig. 6C–I).

To further determine whether the protective effects of QSG were mediated by Lgmn, RCMs were transfected with si-Lgmn and subsequently co-cultured with ACs. Live-cell imaging demonstrated that Lgmn knockdown abolished the QSG-induced enhancement of efferocytosis (Fig. 6J and S7A). Consistently, Lgmn knockdown abolished the upregulatory effects of QSG on Lgmn, Arg1, and Rac1 (Fig. 6K–Q). Collectively, these findings suggest that QSG enhances efferocytosis of RCMs through the Lgmn/Arg1/Rac1 signaling pathway.

### QSG-derived compounds target LGMN to promote RCMs efferocytosis

**Fig. 7:**
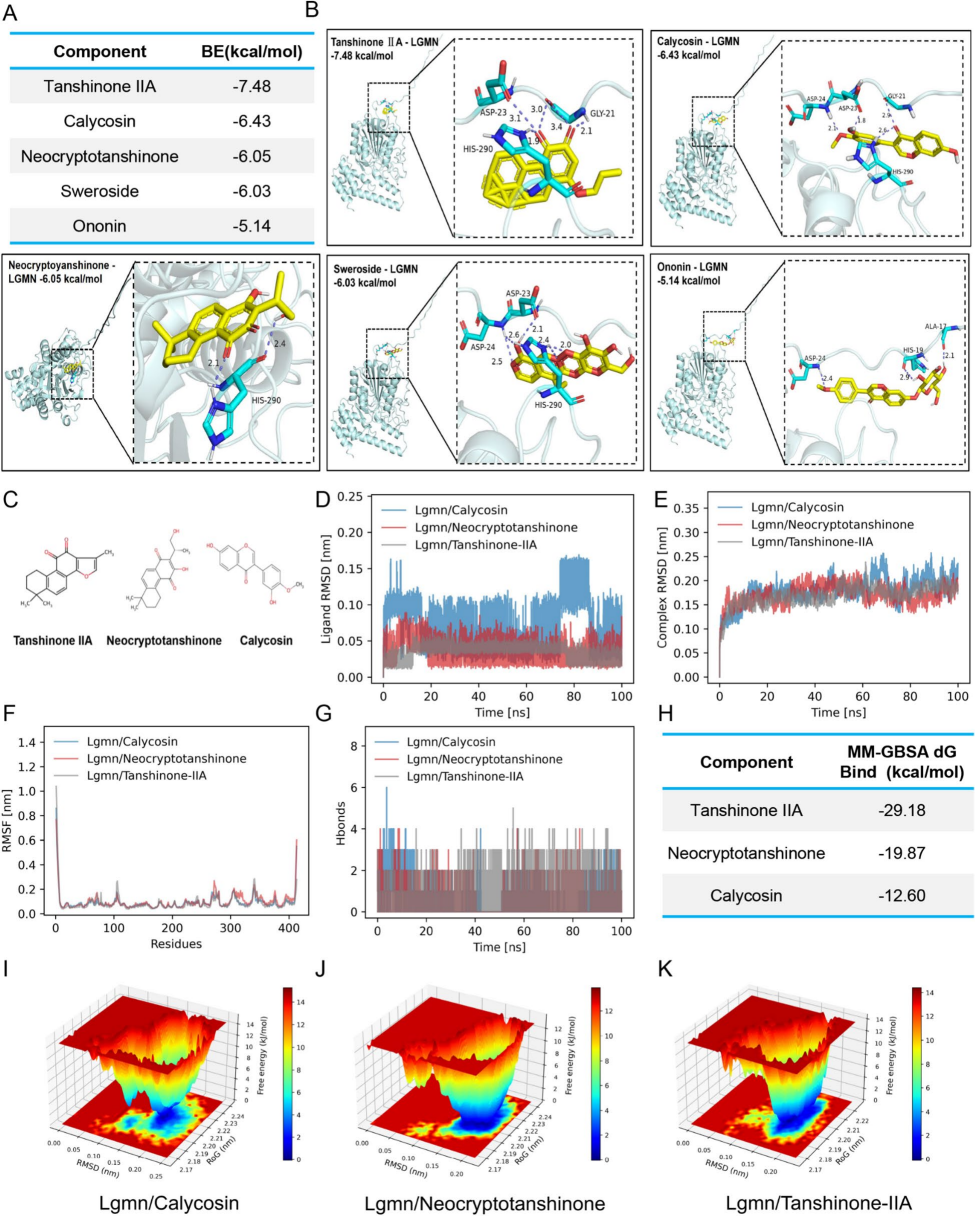
Molecular docking and MDS analysis of the major compounds in QSG. **A** Binding energy results of molecular docking. **B** Representative structures of the protein–ligand complexes. **C** Chemical structures of the top three candidate compounds with the strongest binding affinity to LGMN. **D** RMSD of the ligands. **E** RMSD of the protein–ligand complex. **F** RMSF plot of the protein–ligand complex. **G** Hydrogen bond numbers of the protein–ligand complex. **H** MM-GBSA-calculated binding free energies (kcal/mol) of the three complexes. **I**–**K** Free energy landscapes of three protein–ligand complexs

Molecular docking was performed to evaluate the interactions between major QSG-derived compounds and LGMN. The binding energies (BE) of tanshinone IIA, calycosin, neocryptotanshinone, sweroside, and ononin were all lower than −5 kcal/mol. Notably, tanshinone IIA, calycosin, and neocryptotanshinone exhibited binding energies below −6 kcal/mol, indicating relatively high binding affinities to LGMN (Fig. 7A, B). Given that molecular docking cannot capture the dynamic features of protein–ligand complexes, MDS were further conducted for the top three ligands identified from the docking results. All three ligands formed stable complexes with LGMN, among which neocryptotanshinone and tanshinone IIA exhibited greater ligand stability and lower overall complex fluctuations compared with calycosin (Fig. 7C–G). Binding free energy calculations using the MM/PBSA method further supported strong interactions between these ligands and LGMN (Fig. 7H). Further energy distribution analysis showed that the LGMN/neocryptotanshinone and LGMN/tanshinone IIA complexes exhibited a single deep energy well, with their lowest-energy regions showing a concentrated and continuous distribution (Fig. 7I–K), indicating stable conformational states with limited fluctuations.

**Fig. 8:**
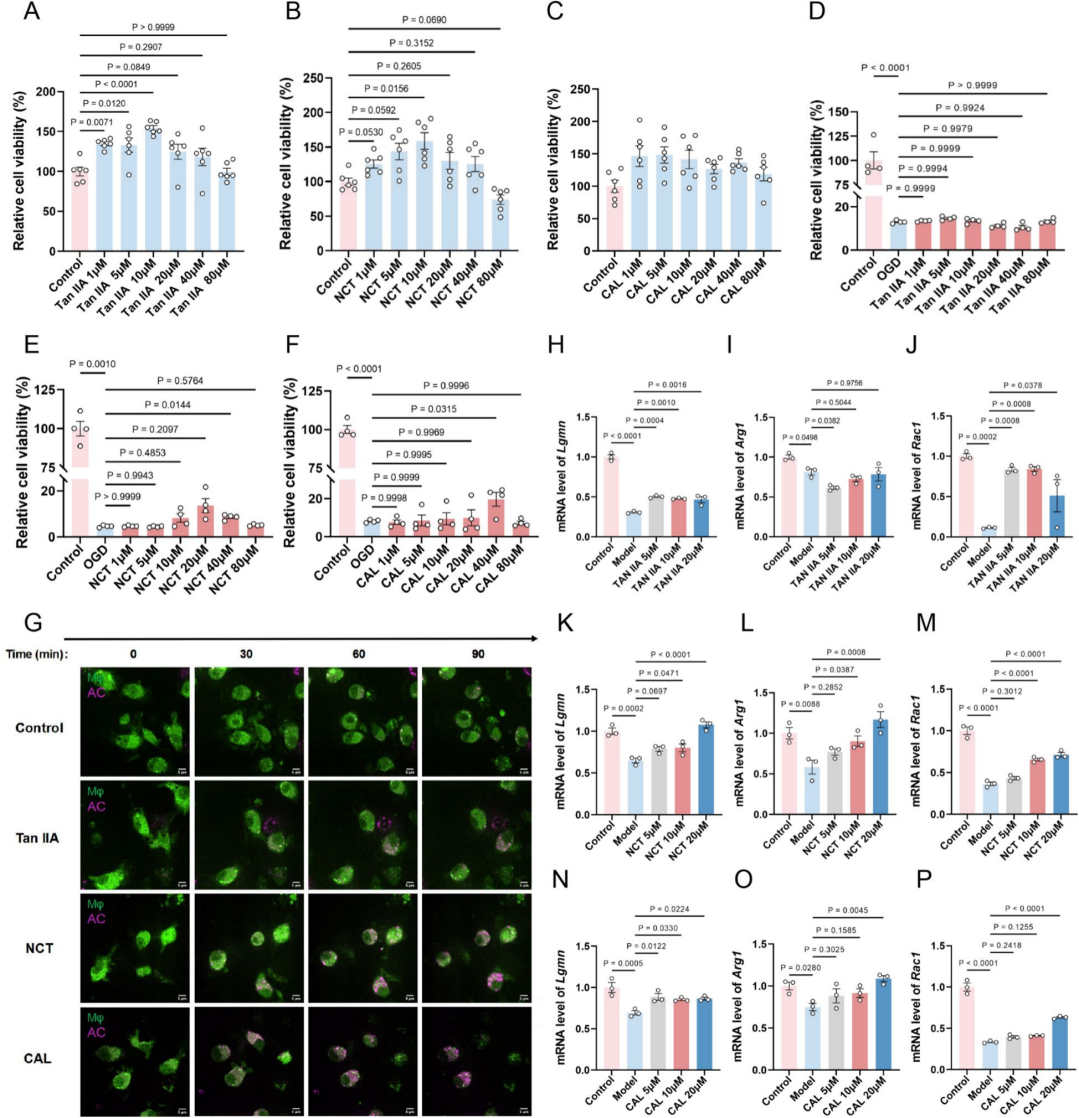
QSG-derived compounds target LGMN to promote RCMs efferocytosis. **A**–**C** CCK-8 assay of RCMs treated with different concentrations of Tan IIA, NCT, and CAL (n = 6). **D**–**F** CCK-8 assay evaluating the protective effects of Tan IIA, NCT, and CAL on RCMs (n = 4). **G** Live-cell imaging of RCMs-ACs co-culture system under different treatment conditions, RCMs (green), ACs (purple), scale bar: 5 μm. **H**–**J** mRNA expression levels of *Lgmn*, *Arg1*, and *Rac1* in RCMs-ACs co-culture system treated with different concentrations of Tan IIA (n = 3). **K**–**M** mRNA expression levels of *Lgmn*, *Arg1*, and *Rac1* in RCMs-ACs co-culture system treated with different concentrations of NCT (n = 3). **N**–**P** mRNA expression levels of *Lgmn*, *Arg1*, and *Rac1* in RCMs-ACs co-culture system treated with different concentrations of CAL (n = 3). n represents the number of independent experiments. All data are presented as mean ± SEM

To validate their biological activities, the effects of these compounds on RCMs viability were assessed using the CCK-8 assay. The results showed that tanshinone IIA, calycosin, and neocryptotanshinone exhibited no cytotoxicity to RCMs at concentrations below 80 μM (Fig. 8A–C). Under oxygen–glucose deprivation (OGD) conditions, treatment with calycosin or neocryptotanshinone significantly improved RCMs viability, whereas tanshinone IIA showed no significant protective effect (Fig. 8D–F). Live-cell imaging further demonstrated that treatment with tanshinone IIA, neocryptotanshinone and calycosin enhanced the efferocytic capacity of RCMs (Fig. 8G and S7B). Furthermore, the effects of these compounds on Lgmn-mediated efferocytosis in RCMs were evaluated by RT-qPCR. These results demonstrated that calycosin and neocryptotanshinone significantly reversed the downregulation of *Lgmn*, *Arg1*, and *Rac1*, whereas tanshinone IIA increased the expression of *Lgmn* and *Rac1* but showed no significant effect on *Arg1* expression (Fig. 8H–P). Collectively, these findings indicate that tanshinone IIA, neocryptotanshinone and calycosin are key bioactive compounds in QSG that promote RCMs efferocytosis by targeting the Lgmn/Arg1/Rac1 signaling axis.

## Discussion

**Fig. 9:**
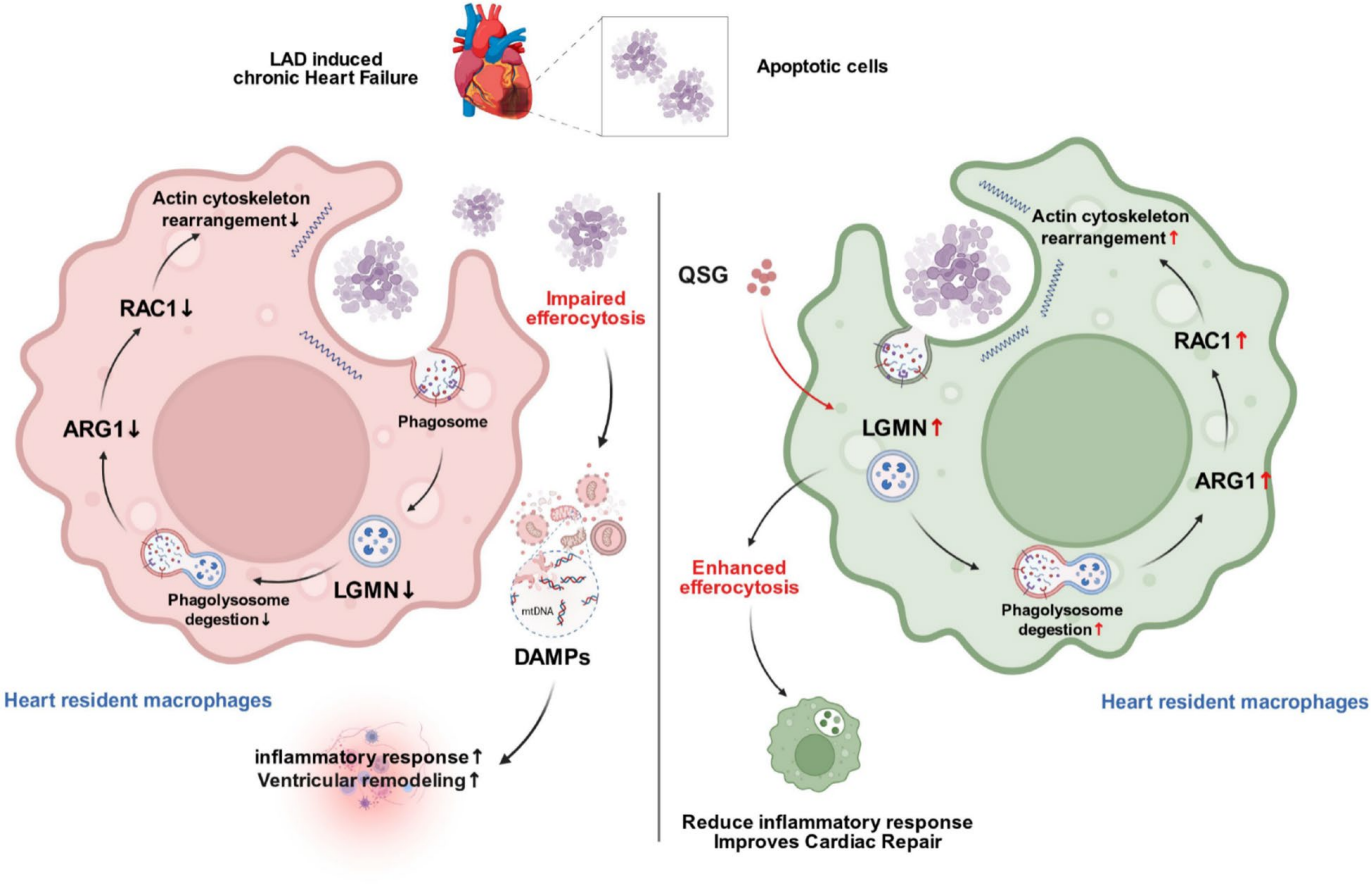
Schematic diagram of the mechanism of QSG in treating CHF

In this study, we systematically evaluated the therapeutic effect of QSG on ventricular remodeling in CHF. Our findings demonstrate that QSG increases the number of RCMs and facilitates the clearance of ACs post-MI by promoting Lgmn/Arg1/Rac1-mediated efferocytosis in RCMs. This effect consequently attenuates adverse ventricular remodeling and improves cardiac function in CHF mice (Fig. 9).

Ventricular remodeling is a major pathophysiological process following MI and a major contributor to the development of CHF [24, 25]. In clinical practice, acute-phase management following MI focuses on limiting myocardial injury and reducing mortality. Once the condition stabilizes after approximately 7 days, treatment transitions to the recovery phase, shifting focus toward promoting myocardial repair and preventing CHF progression. Our previous studies have demonstrated that QSG exerts significant therapeutic effects in CHF. However, previous interventions have primarily targeted the acute phase of MI, with relatively limited attention paid to the recovery phase—a stage that more closely aligns with actual clinical practice. Inadequate myocardial repair and residual ACs are key factors in heart failure progression following MI [24, 26]. Accordingly, in the present study, QSG was administered to mice with CHF from days 7 to 14 after LAD ligation. We demonstrated that QSG treatment during this window significantly improved cardiac function, alleviated myocardial injury, and inhibited adverse ventricular remodeling. To further elucidate the protective mechanisms of QSG in CHF, we performed integrated network pharmacology analysis, identifying potential pathways, including apoptosis, efferocytosis and lysosomal function. These findings suggest that QSG may exert its therapeutic effects through these pathways. Consistently, we observed that QSG reduced the number of ACs in CHF mice, downregulated cleaved caspase-3 and Bax expression, and upregulated Bcl-2 expression. These findings suggest that the cardioprotective effects of QSG are associated not only with suppression of apoptosis, but also with enhanced clearance of ACs.

Macrophages are the major immune cell population in the myocardium following injury and play pivotal regulatory roles throughout all phases post-MI [27, 28]. Cardiac macrophages are mainly composed of two different subpopulations: CCR2^+^ macrophages derived from monocytes and CCR2^−^ RCMs derived from the yolk sac [5]. Following MI, extensive cardiomyocyte death leads to the release of damage-associated molecular patterns (DAMPs), which activate Toll-like receptor signaling and trigger a robust inflammatory response [29]. However, the inflammatory responses mediated by monocyte-derived macrophages are often prolonged or excessive. Therefore, limiting the duration of the inflammatory phase and facilitating the transition to the anti-inflammatory phase are critical for improving long-term outcomes after MI [30]. In contrast, RCMs play essential roles in maintaining immune homeostasis and promoting tissue repair [31, 32]. These cells efficiently clear debris and necrotic cells, and secrete anti-inflammatory mediators such as TGF-β via efferocytosis, thereby promoting inflammation resolution, facilitating cardiac repair, and mitigating ventricular remodeling [33, 34]. Previous studies have demonstrated a marked reduction in RCMs following MI, which may impair efferocytosis and delay tissue repair [35]. Consistently, we observed a decreased proportion of MHC-II^low^CCR2^−^ RCMs in the hearts of CHF mice at 14 days post-MI, while QSG treatment significantly increased this proportion. Notably, CCR2^−^ RCMs expressing MERTK exhibit greater phagocytic capacity for myocardial debris than CCR2^+^ macrophages. Accordingly, we further investigated whether QSG enhances efferocytosis in CHF mice. Our results showed that QSG treatment upregulates key efferocytosis-related signaling molecules, including Tim4, S1p, and Cx3cr1. Collectively, these findings suggest that the therapeutic effects of QSG are associated with enhanced RCMs-mediated efferocytosis.

Lgmn is a lysosomal cysteine protease that plays a critical role in intracellular protein processing and macrophage function [7, 36]. Accumulating evidence indicates that Lgmn deficiency markedly impairs macrophage-mediated phagocytosis of ACs, cellular debris, and lipoproteins in vivo and in vitro, supporting an important role for Lgmn in efferocytosis. Recent studies have further identified Lgmn as a key lysosomal enzyme involved in efferocytosis and highly expressed in RCMs after MI. Mechanistically, Lgmn promotes protease-activated receptor 2 cleavage, leading to sustained intracellular Ca^2+^ signaling in macrophages, which facilitates sustained efferocytosis and improves cardiac function [37]. In addition, delivery of *Lgmn* mRNA to chimeric antigen receptor-engineered macrophages (CAR-MΦ) has been shown to enhance efferocytosis capacity and alleviate cardiac fibrosis, further supporting its therapeutic potential [38]. In the present study, transcriptomic analysis of cardiac macrophages from CHF mice at day 7 post-MI revealed that Lgmn was significantly upregulated and closely associated with efferocytosis-related pathways. Subsequently, through integrated analysis of network pharmacology and transcriptomics, we identified Lgmn as a potential key target through which QSG ameliorates CHF by modulating efferocytosis. Notably, temporal profiling demonstrated that Lgmn expression peaked at day 7 post-MI and subsequently declined, suggesting that Lgmn may play an important role during the early reparative phase after MI (Fig. S8A, B). Based on these findings, we hypothesized that impaired efferocytosis in CHF is associated with reduced RCM abundance and decreased Lgmn expression. Consistent with this hypothesis, QSG treatment significantly upregulated Lgmn expression in RCMs, supporting a mechanism whereby QSG enhances efferocytosis through Lgmn upregulation.

Previous studies have shown that macrophage Arg1 metabolizes arginine derived from ACs into ornithine, which contributes to Rac1-mediated actin cytoskeletal remodeling [4, 39]. This process enhances sustained efferocytosis and ACs clearance. Transcriptomic analysis of cardiac macrophages from Lgmn knockout mice revealed markedly reduced Arg1 expression, suggesting that Lgmn may regulate efferocytosis of RCMs through the Arg1/Rac1 axis. Consistent with these findings, our in vivo and in vitro experiments demonstrated that QSG enhances RCMs-mediated efferocytosis by activating the Lgmn/Arg1/Rac1 signaling pathway. Collectively, these findings indicate that Lgmn may regulate efferocytosis through a dual mechanism: on the one hand, it enhances post-engulfment degradation via its enzymatic activity; on the other hand, it promotes Rac1-dependent actin cytoskeletal remodeling required for efferocytosis through activation of the Arg1/Rac1 signaling axis. This dual mechanism provides a coherent explanation for the sustained enhancement of efferocytosis observed following QSG treatment.

This study conducted a comprehensive analysis of the major bioactive components in QSG. Molecular docking and MDS suggested that tanshinone IIA, neocryptotanshinone and calycosin exhibit strong binding affinity to LGMN, which are derived respectively from “Monarch Drug” and “Minister Drug” of QSG. Previous studies have confirmed their therapeutic effects on ischemic heart disease. In this study, RT-qPCR analysis showed that calycosin and neocryptotanshinone upregulated the expression of Lgmn, Arg1, and Rac1, whereas tanshinone IIA increased Lgmn and Rac1 expression but had no significant effect on Arg1 in RCMs. However, CCK-8 assays revealed that tanshinone IIA did not significantly improve the viability of RCMs under OGD conditions. Taken together, these findings indicate that tanshinone IIA primarily modulates macrophage function, particularly efferocytosis-related activity, rather than directly improving cell viability under OGD conditions. This interpretation is consistent with previous studies reporting that tanshinone IIA mainly regulates macrophage phenotypic polarization [40, 41]. Collectively, tanshinone IIA, neocryptotanshinone, and calycosin are key bioactive compounds contributing to the therapeutic effects of QSG [42, 43]. However, the potential synergistic interactions among multiple components within QSG warrant further investigation. Several limitations should be acknowledged. First, the experimental observations were limited to two-week post-MI assessments and did not evaluate long-term outcomes, including sustained cardiac function, survival, or arrhythmia-related events. Second, the current research predominantly focused on RCMs, while the effects of QSG on other immune cells such as dendritic cells and T cells, as well as their intercellular crosstalk with macrophages in CHF, remain unclear. In addition, Rac1-GTP levels were not directly measured, limiting the definitive assessment of Rac1 activation and its mechanistic contribution to QSG effects. These limitations will be further addressed in future studies.

## Conclusion

In summary, this study demonstrates that QSG attenuates adverse ventricular remodeling in CHF by enhancing efferocytosis in RCMs. Mechanistically, QSG exerts its protective effects through activation of the Lgmn/Arg1/Rac1 signaling axis, thereby promoting ACs clearance and facilitating cardiac repair. Furthermore, neocryptotanshinone and calycosin were identified as key bioactive compounds that target Lgmn and enhance efferocytosis by promoting Arg1/Rac1-mediated cytoskeletal remodeling. Collectively, these findings provide valuable insights into the clinical application of QSG for CHF treatment and highlight RCMs-mediated efferocytosis as a potential therapeutic target for chronic heart failure.

## Supplementary Information

Supplementary Material 1.

## Data Availability

Data will be made available on request.

## Abbreviations

ACs: Apoptotic cells
Arg1: Arginase 1
CHF: Chronic heart failure
GO: Gene Ontology
KEGG: Kyoto Encyclopedia of Genes and Genomes
LAD: Left anterior descending coronary artery
Lgmn: Legumain
MDS: Molecular dynamics simulations
MI: Myocardial infarction
QSG: Qishen Granules
QSG-H: High-dose QSG
QSG-L: Low-dose QSG
Rac1: Ras-related C3 botulinum toxin substrate 1
RCMs: Resident cardiac macrophages
SD: Sprague–Dawley
