# Supplementary material for: Qishen Granules attenuate adverse ventricular remodeling in chronic heart failure by promoting Legumain/Arg1/Rac1-mediated efferocytosis of resident cardiac macrophages
Source: Chin Med. 2026 Jul 8;21:184. doi: 10.1186/s13020-026-01461-6 (PMC13343700; doi:10.1186/s13020-026-01461-6)
Supplement: Supplementary file 1 — Supplementary Material 1. [file 13020_2026_1461_MOESM1_ESM.docx]

**Supplementary data**

**Figure S1**


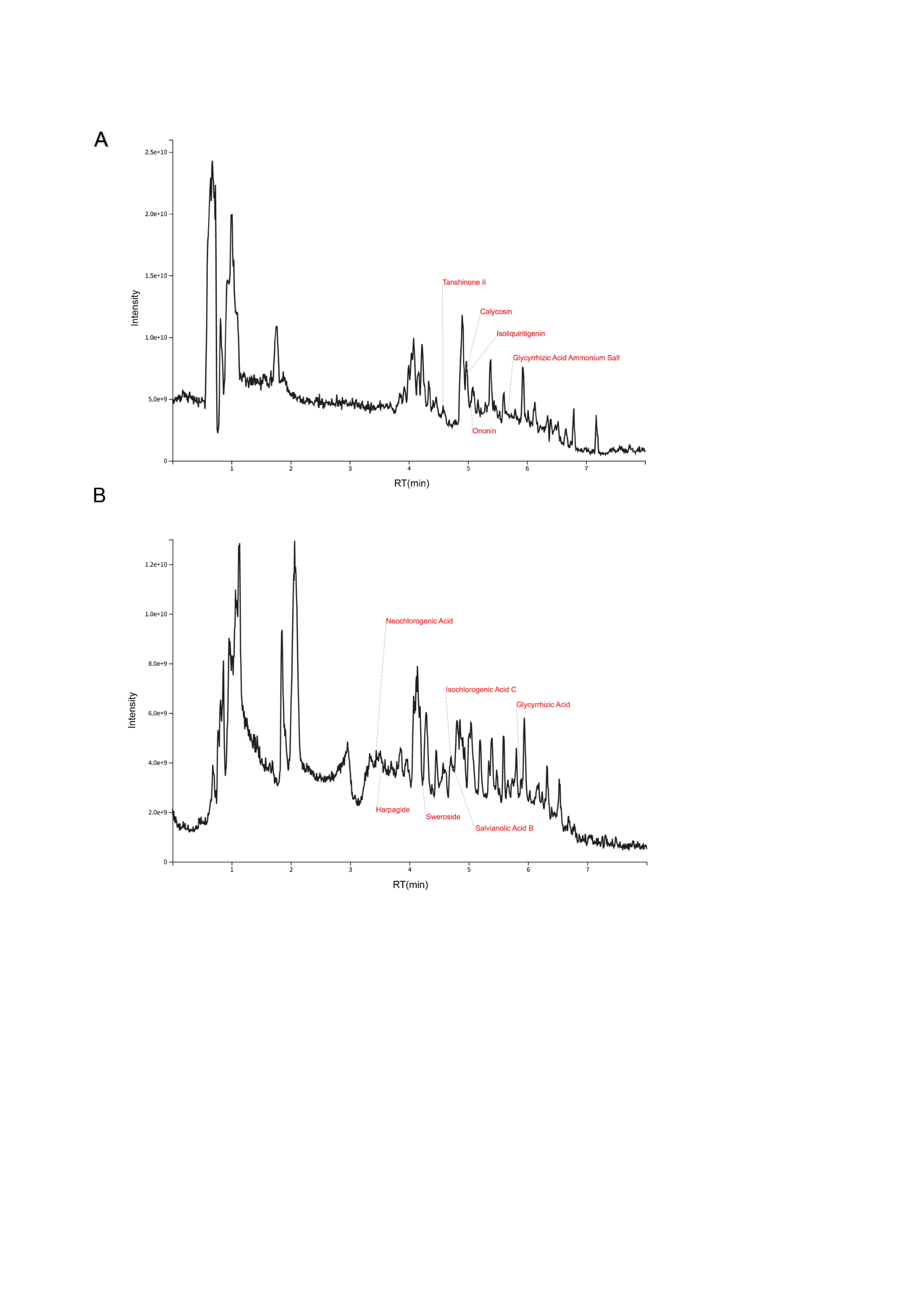


Figure S1. Identification of the components of QSG. (A) Total Ion Chromatogram (TIC) of compounds in QSG under positive ion mode. (B) TIC of compounds in QSG under negative ion mode.

**Figure S2**


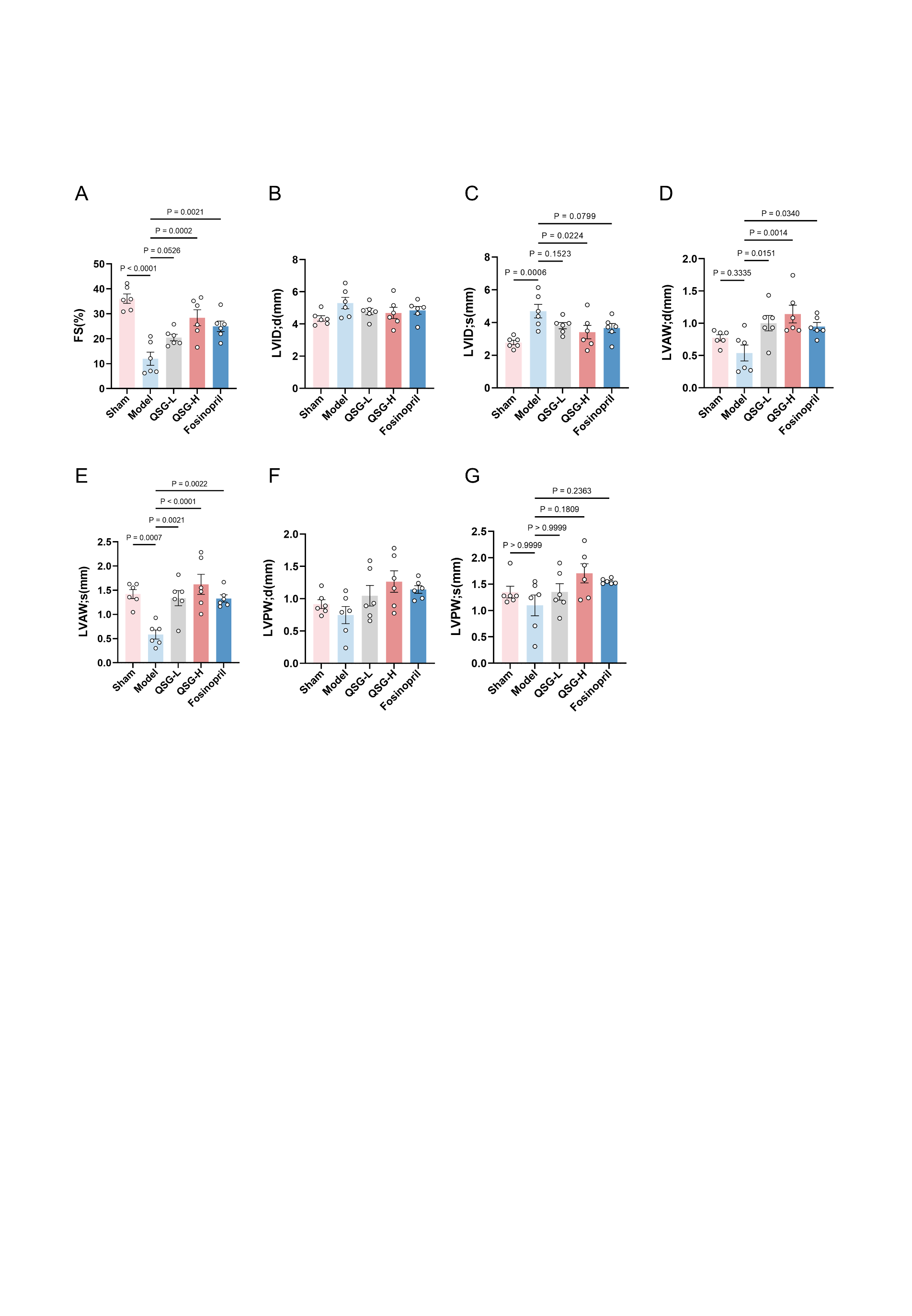


Figure S2. Effects of QSG treatment on echocardiographic parameters in heart failure mice. (A) FS of mice in each group (n=6). (B) LVID;d of mice in each group (n=6). (C) LVID;s of mice in each group (n=6). (D) LVAW;d of mice in each group (n=6). (E) LVAW;s of mice in each group (n=6). (F) LVPW;d of mice in each group (n=6). (G) LVPW;s of mice in each group (n=6). n indicates the number of experimental animals per group. All data are presented as mean ± SEM.

**Figure S3**


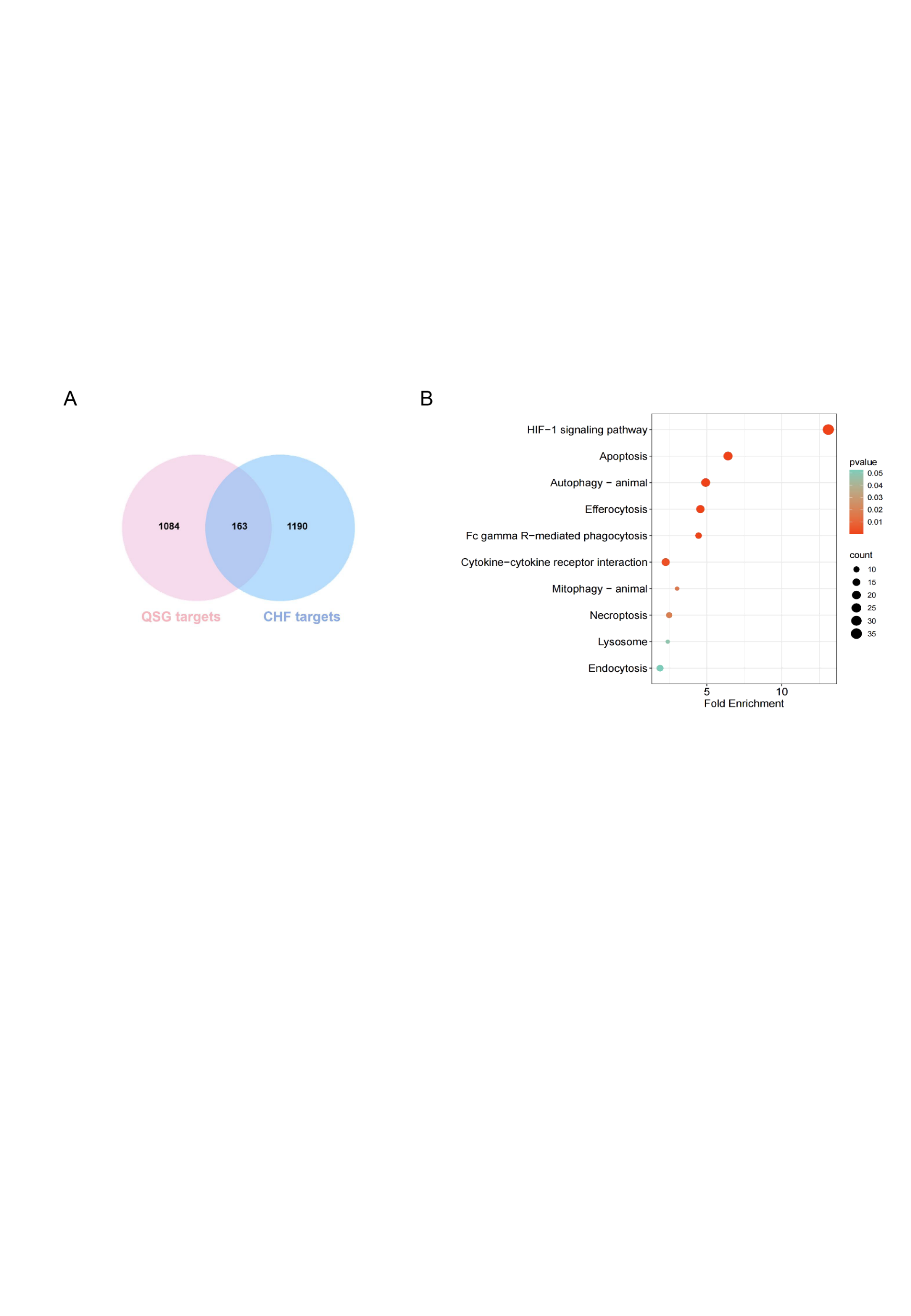


Figure S3. Network pharmacology analysis of QSG in the treatment of CHF. (A) Venn diagram of intersection targets between CHF and QSG. (B) KEGG pathway enrichment analysis of the overlapping targets.

**Figure S4**


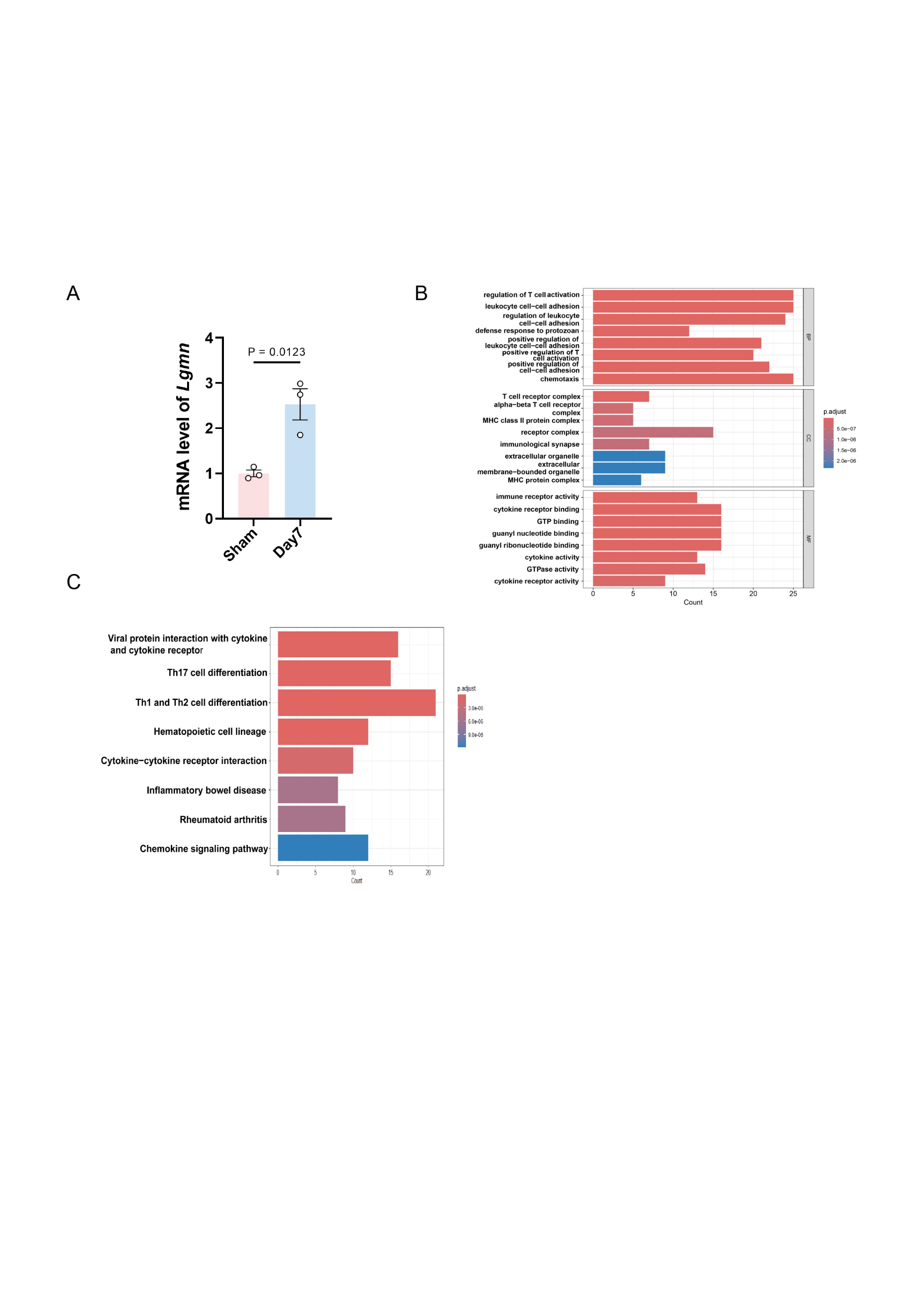


Figure S4. (A) Relative *Lgmn* mRNA expression at day 7 post‑MI (n=3). (B) Top 8 GO terms from mRNA sequencing analysis of cardiac macrophages in WT and Lgmn^-/-^ mice after MI. (C) Top 8 KEGG pathways from mRNA sequencing analysis of cardiac macrophages in WT and Lgmn^-/-^ mice after MI. n indicates the number of experimental animals per group. All data are presented as mean ± SEM.

**Figure S5**


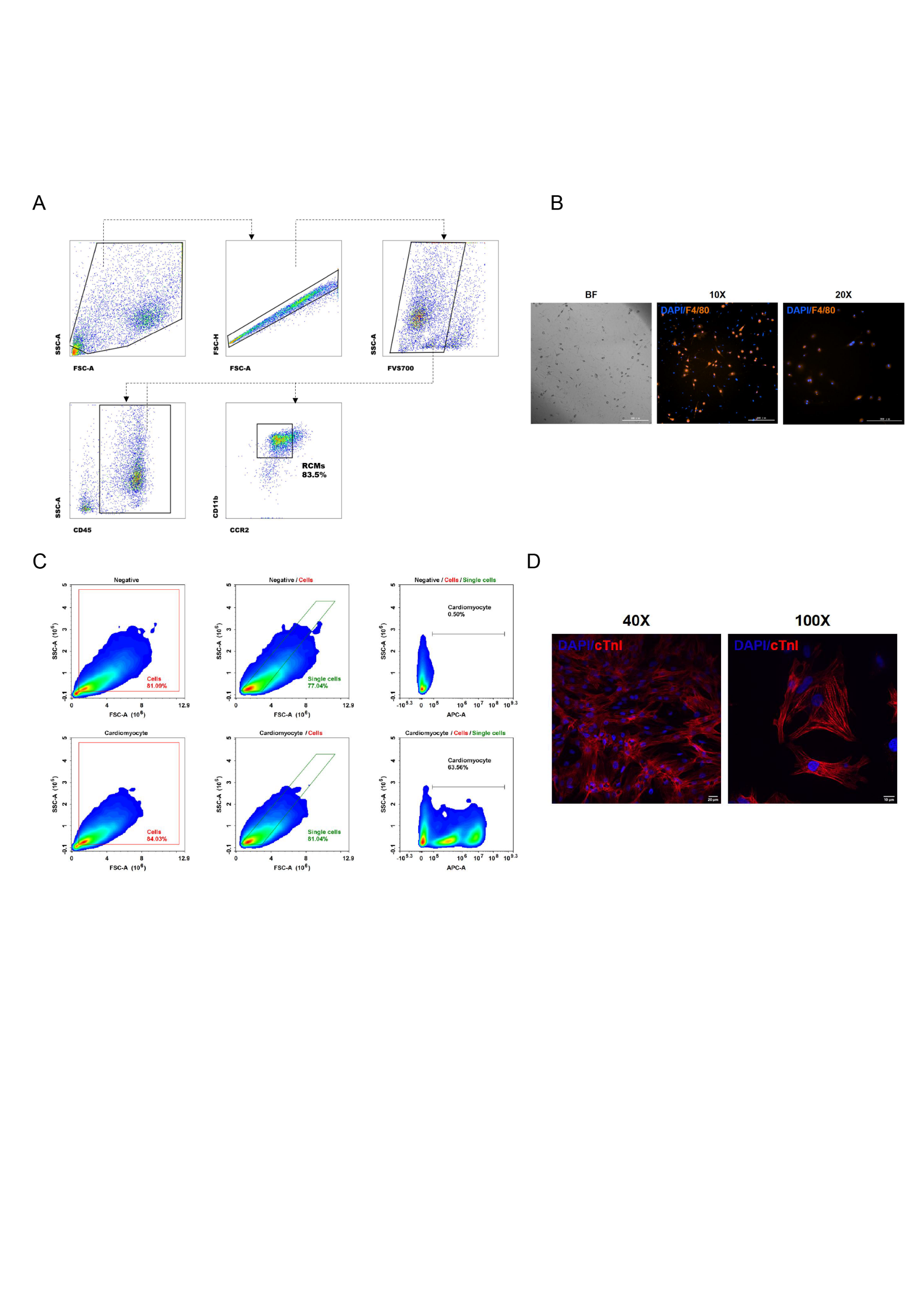


Figure S5. Identification of RCMs and primary cardiomyocytes. (A) Flow cytometry gating plots for RCMs. (B) Representative brightfield and immunofluorescence images of RCMs, F4/80 (orange), DAPI (blue), scale bars: 300 μm and 200 μm. (C) Flow cytometry gating plots for primary cardiomyocytes. (D) Representative immunofluorescence images of primary cardiomyocytes, cTnI (red), DAPI (blue), scale bar: 20 μm and 10 μm.

**Figure S6**


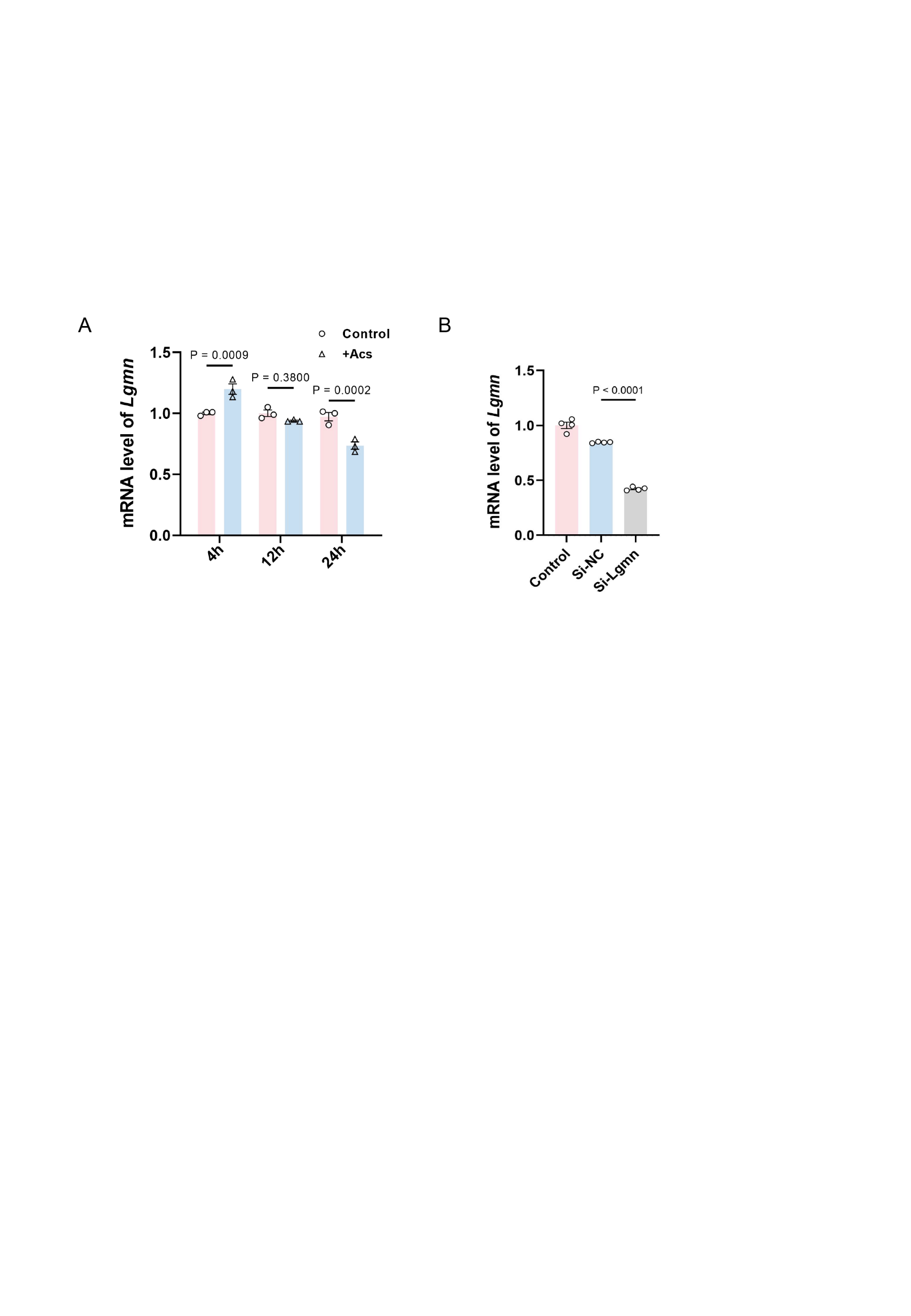


Figure S6. (A) mRNA expression levels of *Lgmn* in RCMs-ACs co-culture at 4 h, 12 h, and 24 h (n=3). (B) mRNA expression level of *Lgmn* after Lgmn knockdown (n=4). n represents the number of independent experiments. All data are presented as mean ± SEM.

**Figure S7**


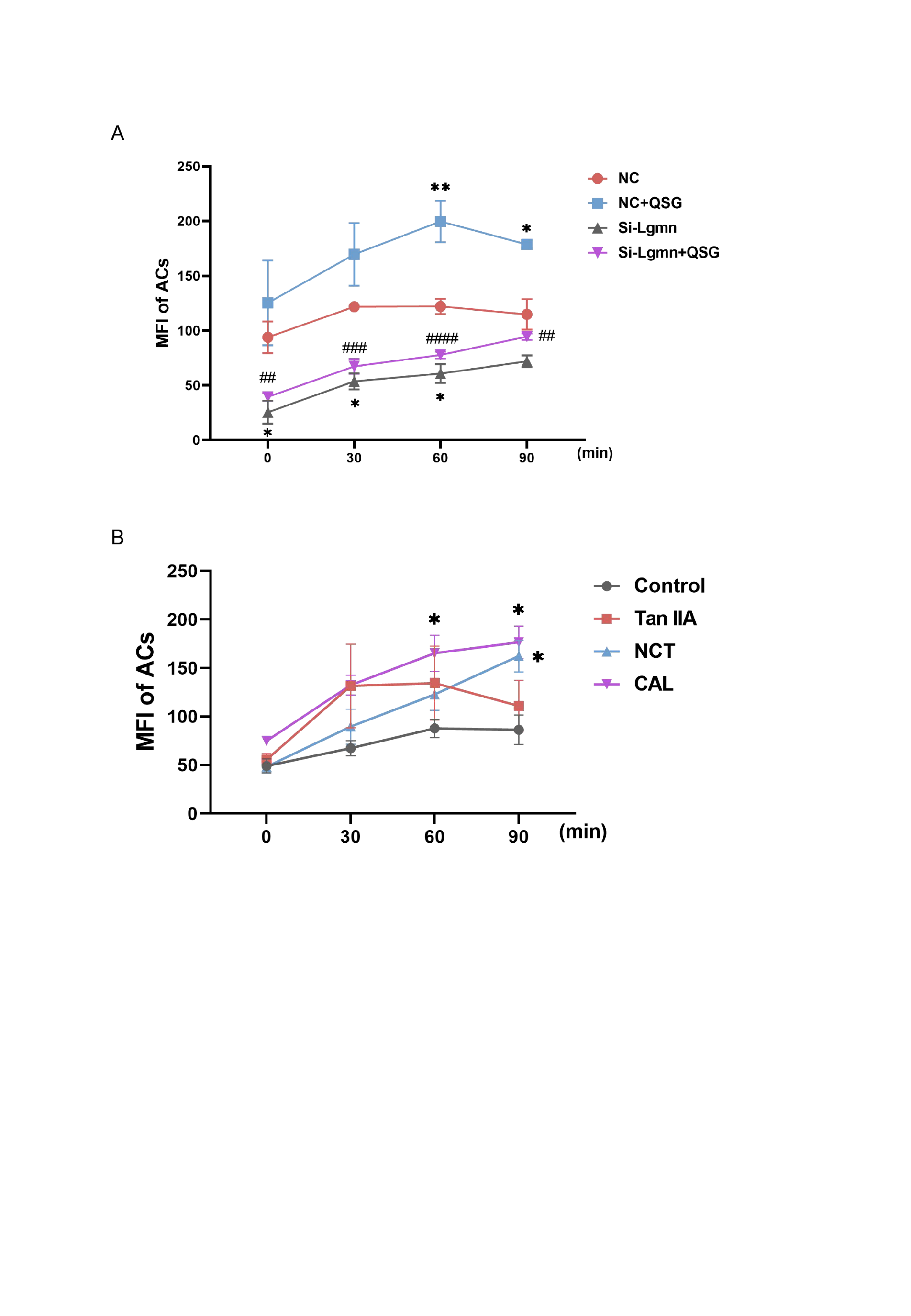


Figure S7. (A) Quantitative analysis of efferocytosis in RCMs using live-cell imaging (n=3). **P* < 0.05, ***P* < 0.01 (vs. NC at the same time point). ##*P* < 0.01, ###*P* < 0.001, ####*P* < 0.0001 (vs. NC + QSG at the same time point). (B) Quantitative analysis of efferocytosis in RCMs using live-cell imaging (n=3). **P* < 0.05 (vs. the Control at the same time point). n represents the number of independent experiments. All data are presented as mean ± SEM.

**Figure S8**


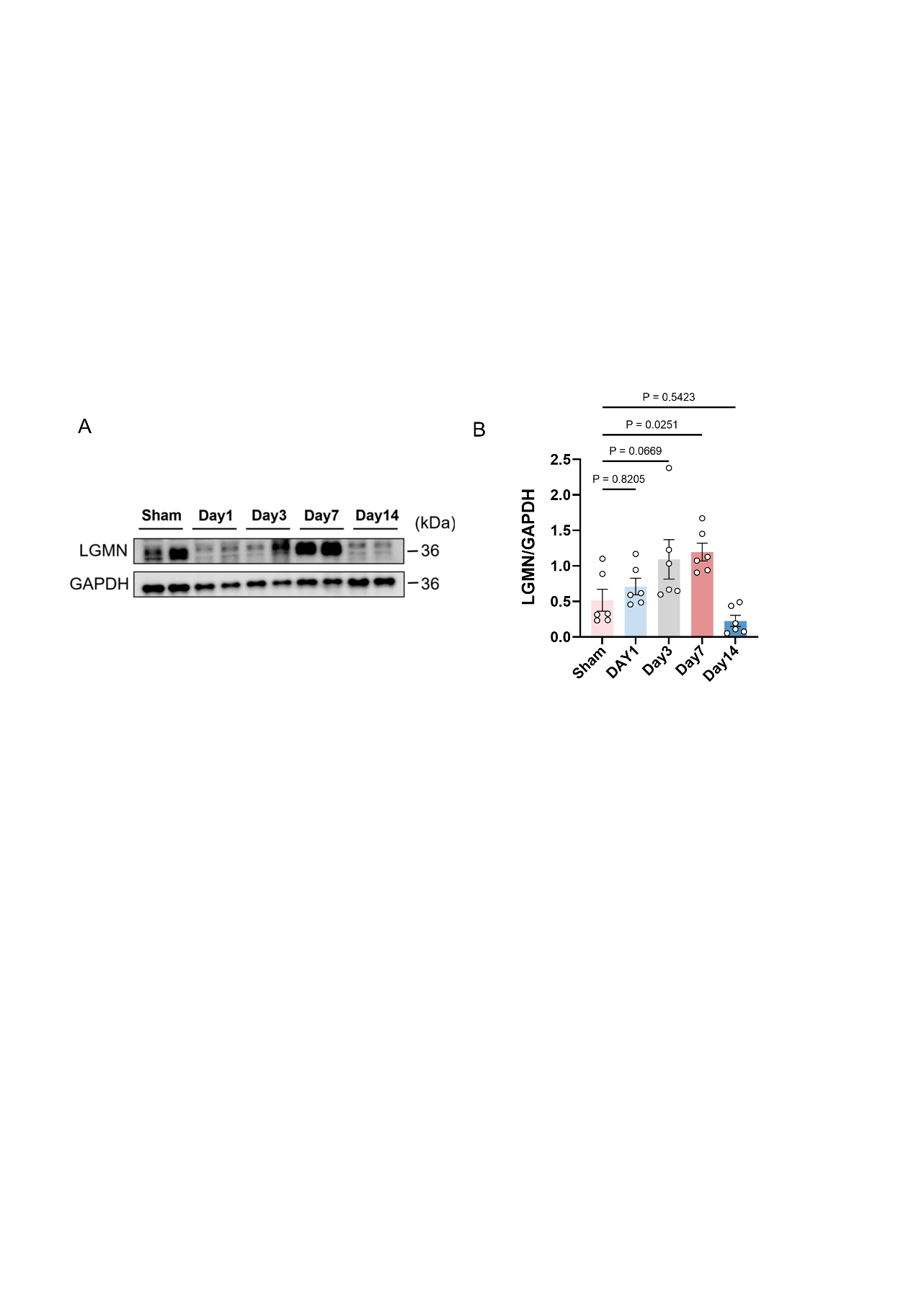


Figure S8. Expression of LGMN at Different Time Points Following MI. (A) Representative Western blot image of LGMN at different time points after MI-induced heart failure. (B) Quantitative analysis of LGMN protein expression by Western blot at different time points after MI-induced heart failure (n=6). n indicates the number of experimental animals per group. All data are presented as mean ± SEM.
